# Aculeaxanthones A–E, new xanthones from the marine-derived fungus *Aspergillus aculeatinus* WHUF0198

**DOI:** 10.3389/fmicb.2023.1138830

**Published:** 2023-02-27

**Authors:** Jun Wu, Hua Shui, Mengke Zhang, Yida Zeng, Mingxin Zheng, Kong-Kai Zhu, Shou-Bao Wang, Hongkai Bi, Kui Hong, You-Sheng Cai

**Affiliations:** ^1^Department of Nephrology, Zhongnan Hospital of Wuhan University, School of Pharmaceutical Sciences, Wuhan University, Wuhan, China; ^2^Key Laboratory of Combinatorial Biosynthesis and Drug Discovery, Ministry of Education and School of Pharmaceutical Sciences, Wuhan University, Wuhan, China; ^3^Department of Pharmacy, Renmin Hospital of Wuhan University, Wuhan, China; ^4^Department of Pathogen Biology & Jiangsu Key Laboratory of Pathogen Biology & Helicobacter pylori Research Centre, Nanjing Medical University, Nanjing, China; ^5^Advanced Medical Research Institute, Shandong University, Jinan, Shandong, China; ^6^Beijing Key Laboratory of Drug Targets Identification and Drug Screening, Institute of Materia Medica, Chinese Academy of Medical Sciences and Peking Union Medical College, Beijing, China

**Keywords:** *Aspergillus aculeatinus*, dimeric tetrahydroxanthones, marine natural products, antimicrobial activity, cytotoxic activity

## Abstract

**Introduction:**

Dimeric natural products are widespread in plants and microorganisms, which usually have complex structures and exhibit greater bioactivities than their corresponding monomers. In this study, we report five new dimeric tetrahydroxanthones, aculeaxanthones A−E (**4−8**), along with the homodimeric tetrahydroxanthone secalonic acid D (**1**), chrysoxanthones B and C (**2** and **3**), and 4−4’-secalonic acid D (**9**), from different fermentation batches of the title fungus.

**Methods:**

A part of the culture was added to a total of 60 flasks containing 300 ml each of number II fungus liquid medium and culture 4 weeks in a static state at 28˚C. The liquid phase (18 L) and mycelia was separated from the fungal culture by filtering. A crude extract was obtained from the mycelia by ultrasound using acetone. To obtain a dry extract (18 g), the liquid phase combined with the crude extract were further extracted by EtOAc and concentrated in vacuo. The MIC of anaerobic bacteria was examined by a broth microdilution assay. To obtain MICs for aerobic bacteria, the agar dilution streak method recommended in Clinical and Laboratory Standards Institute document (CLSI) M07-A10 was used. Compounds 1−9 was tested against the Bel-7402, A-549 and HCT-116 cell lines according to MTT assay.

**Results and Discussion:**

The structures of these compounds were elucidated on the base of 1D and 2D NMR and HR-ESIMS data, and the absolute configurations of the new xanthones **4−8** were determined by conformational analysis and time-dependent density functional theory-electronic circular dichroism (TDDFT-ECD) calculations. Compounds 1–9 were tested for cytotoxicity against the Bel-7402, A549, and HCT-116 cancer cell lines. Of the dimeric tetrahydroxanthone derivatives, only compound 6 provided cytotoxicity effect against Bel-7402 cell line (IC50, 1.96 µM). Additionally, antimicrobial activity was evaluated for all dimeric tetrahydroxanthones, including four Gram-positive bacteria including Enterococcus faecium ATCC 19434, *Bacillus subtilis* 168, *Staphylococcus aureus* ATCC 25923 and MRSA USA300; four Gram-negative bacteria, including *Helicobacter pylori* 129, G27, as well as 26,695, and multi drug-resistant strain *H. pylori* 159, and one *Mycobacterium M. smegmatis* ATCC 607. However, only compound 1 performed activities against *H. pylori* G27, *H. pylori* 26695, *H. pylori* 129, *H. pylori* 159, *S. aureus* USA300, and *B. subtilis* 168 with MIC values of 4.0, 4.0, 2.0, 2.0, 2.0 and 1.0 μg/mL, respectively.

## Introduction

Dimeric natural products are widespread in plants and microorganisms, which usually have complex structures and exhibit greater bioactivities than their corresponding monomers (Wezeman et al., 2015; Cai et al., 2018; Lombe et al., 2019). In fungi and lichens, dimeric tetrahydroxanthones (synonyms ergochromes, ergopigments, ergoflavins, ergochrysins, secalonic acids) are important polyketides with diverse structures (Franck, 1969, 1980; Rezanka and Sigler, 2007; Deshmukh et al., 2009). The dimeric tetrahydroxanthones are usually classified as heterodimer and homodimer, with 2–2′, 2–4′, or 4–4′ linkage according to the structural differences of two monomers (Wezeman et al., 2015). The homodimers are composed of the same two chromanone lactone subunits (a γ-lactone moiety linked to a dihydrobenzopyranone) or two tetrahydroxanthone monomers, while the heterodimers consist of two different tetrahydroxanthone monomers and/or chromanone monomers or their derivatives (Zhang et al., 2008; El-Elimat et al., 2015). Due to their interesting chemical properties and a broad spectrum of bioactivities, for instance, antimicrobial, antiviral, anti-inflammatory, and antiparasitic activities as well as cytotoxicity (Cai et al., 2014; Wu et al., 2015; Luenne et al., 2021; Sadorn et al., 2021; Cao et al., 2022; Phang et al., 2022), dimeric tetrahydroxanthones have been extensively studied in chemical and pharmacological fields (Masters and Brase, 2012; Roensberg et al., 2013; Qin et al., 2015a; Qin and Porco, 2015; Xiao et al., 2017; Lv et al., 2021).

In the process of our ongoing screening for new biologically active natural products from marine-derived fungi, we discovered that the fungus *Aspergillus aculeatinus* WHUF0198 contained an assortment of chemically diverse metabolites revealed by LC-ESIMS (UV–vis) profiles, and displayed potent antibacterial and antitumor properties during our preliminary screening of bioassays. We previously reported one new norditerpene, one new indone, and one paraherquamide alkaloid, along with 13 known compounds from the culture of this fungus (Wu et al., 2021, 2022). In this study, we report five new dimeric tetrahydroxanthones, aculeaxanthones A–E (**4**–**8**), along with the homodimeric tetrahydroxanthone secalonic acid D (**1**), chrysoxanthones B and C (**2** and **3**), and 4–4′-secalonic acid D (**9**), from different fermentation batches of the title fungus. In contrast to the reported dimeric tetrahydroxanthones, compounds **4**–**8** have many kinds of dimeric patterns, covering the common 2–2′ linkage (**5**–**7**) and the less prevalent 2–4′ linkage (**4** and **8**). Employing NMR and ECD spectroscopy and TDDFT calculations, the absolute configurations of these tetrahydroxanthones were investigated. What’s more, the cytotoxicity and antibacterial evaluation of all the isolates were also discussed herein.

## Materials and methods

### General experimental procedures

A PerkinElmer Model 341 polarimeter was applied to measure optical rotations. ECD spectra were acquired using a Chirascan V100 spectropolarimeter. NMR data were recorded at 400 or 600 MHz (Bruker AVANCE). HRESIMS spectra were obtained on a ThermoFisher mass spectrometer (LTQ Orbitrap XL). Size-exclusion chromatography was conducted with Sephadex LH-20. Column chromatography (CC) was applied using silica gel which was produced by Anhui Liangchen Co., Ltd.

### Fungal material and mass culture

A specimen of *A. aculeatinus* was identified using the ITS sequences and the morphological characteristics (Wu et al., 2021). A voucher specimen (WHUF0198) has been preserved in School of Pharmaceutical Sciences, Wuhan University. The fungus was precultivated on number II fungus medium (Wu et al., 2022) and incubated at 28°C for a week. After that, a part of the culture was added to a total of 60 flasks containing 300 ml each of number II fungus liquid medium and culture 4 weeks in a static state at 28°C.

### Extraction and isolation

**Figure 1:**
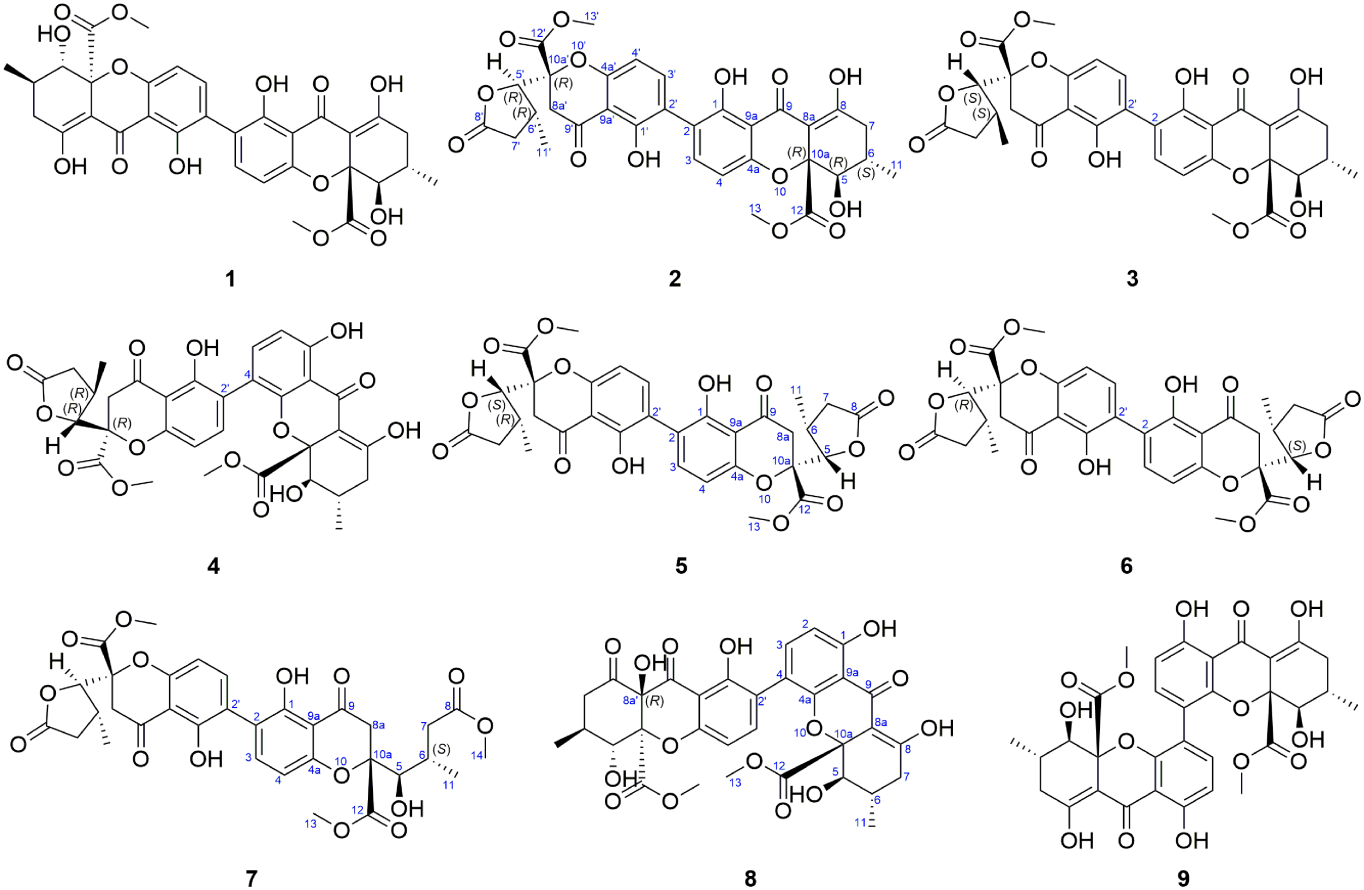
Structures of dimeric tetrahydroxanthones **1**–**9**.

The liquid phase (18 L) and mycelia was separated from the fungal culture by filtering. A crude extract was obtained from the mycelia by ultrasound using acetone. To obtain a dry extract (18 g), the liquid phase combined with the crude extract were further extracted by EtOAc and concentrated *in vacuo*. The dry extract was divided into six fractions (F1–F6) and a silica gel CC was applied to gain twenty-six fractions (F5a–F5z) as described earlier (Wu et al., 2022). F5n (125 mg), F5y (110 mg) and F5z (90 mg) was separated using a Sepex-C18 column (250 × 10 mm, 5 μm) with DAD detection and a flow rate of 3 ml/min to gain **2** (5 mg, 68% MeOH-H_2_O, t_R_ 25.3 min), **8** (5 mg, MeCN/H_2_O, 55:45, v/v, t_R_ 22.3 min) and **9** (2 mg, 75% MeOH-H_2_O, t_R_ 24.2 min). F5i (400 mg) was separated using Sephadex LH-20 (MeOH-CH_2_Cl_2_ 1:1) to yield ten fractions (F5i.1–F5i.10). F5i.5 (30 mg) was repurified by Sepex-C18 column with a shimodzu HPLC system (60–80% MeOH-H_2_O) to yield **3** (2 mg, 3 ml/min, t_R_ 24.8 min), **4** (1 mg, 3 ml/min, t_R_ 26.5 min). F5i.3 (40 mg) was repurified by Sepex-C18 column (65–70% MeOH-H_2_O over 28 min) to gain **5** (1 mg, 3 ml/min, t_R_ 25.6 min), **6** (2 mg, 3 ml/min, t_R_ 18.3 min) and **7** (1 mg, 3 ml/min, t_R_ 27.1 min) (Figure 1).

*Aculeaxanthone A (**4**)*: orange powder; ${\left[\alpha \right]}_{D}^{20}$ +272.5 (*c* 0.04, CDCl_3_); ECD (1.5 × 10^−4^ M, MeOH), *λ* [nm] (Δ*ε*) 292 (+1.68), 246 (−32.51), 223 (−48.65); ^1^H and ^13^C NMR (CDCl_3_), see Table 1; HR-ESIMS (*m/z*): 661.1525 [M + Na]^+^ (calcd for C_32_H_30_O_14_Na, 661.1533).

**Table 1:** ^1^H and ^13^C NMR spectroscopic data for 4 and 8 (CDCl_3_, TMS, *δ* ppm).

No.	**4**		**8**	
	*δ*_C_	*δ*_H_, *J* (Hz)	*δ*_C_	*δ*_H_, *J* (Hz)
1	161.7		158.4	
2	110.4	6.62 d (8.6)	107.7	6.63 d (8.5)
3	140.7	7.49 d (8.6)	140.3	7.47 d (8.5)
4	118.0		117.5	
4a	155.3		159.3	
5	76.9	3.85 d (11.3)	76.8	3.94 d (11.2)
6	29.3	2.40 m	29.2	2.42 dq (11.2, 6.2, 5.6)
7	36.4	2.73 dd (19.2, 6.3)	36.3	2.74 dd (19.2, 6.2)
		2.28 dd (19.2, 10.6)		2.32 dd (19.2, 10.7)
8	177.1		177.7	
8a	101.6		101.5	
9	-		187.1	
9a	107.3		106.9	
10a	85.0		84.8	
11	18.0	1.13 d (6.4)	18.0	1.18 d (6.4)
12	169.9		170.3	
13	53.7	3.79 s	53.3	3.73 s
1-OH		11.42 s		11.77 s
1′	159.6		157.3	
2′	118.0		118.8	
3′	141.6	7.90 d (8.5)	141.5	7.58 dd (8.5, 1.0)
4′	107.2	6.64 d (8.5)	107.1	6.69 dd (8.5, 1.0)
4a′	158.3		160.6	
5′	87.6	4.49 d (3.9)	74.0	4.50 d (10.7)
6′	30.2	2.89 s	32.0	2.09 m
7′	36.0	2.95 dd (17.5, 9.3)	43.4	2.92 dd (15.0, 12.7)
		2.26 dd (17.5, 4.4)		2.51 dd (15.0, 5.3)
8′	175.0		198.5	
8a′	39.9	3.23 d (17.0)	71.8	
		3.11 d (17.0)		
9′	193.8		191.6	
9a′	107.3		106.5	
10a′	84.1		89.4	
11′	20.9	1.32 d (6.8)	18.5	1.22 d (6.3)
12′	169.0		167.9	
13′	53.4	3.70 s	53.6	3.69 s
1′-OH		11.84 s		

*Aculeaxanthone B (**5**)*: yellow powder; ${\left[\alpha \right]}_{D}^{20}$ −9 (*c* 0.03, MeOH); ECD (1.5 × 10^−4^ M, MeOH), *λ* [nm] (Δ*ε*) 264 (−0.91), 223 (+6.02); ^1^H and ^13^C NMR (CDCl_3_), see Table 2; HR-ESIMS (*m/z*): 661.1523 [M + Na]^+^ (calcd for C_32_H_30_O_14_Na, 661.1533).

**Table 2:** ^1^H and ^13^C NMR spectroscopic data for 5–7 (CDCl_3_, TMS, *δ* ppm).

No.	**5**		**6**		**7**	
	*δ*_C_	δ_H_, *J* (Hz)	*δ*_C_	δ_H_, *J* (Hz)	*δ*_C_	*δ*_H_, *J* (Hz)
1	159.7		159.2		158.8	
2	117.4		117.4		117.4	
3	141.4	7.53 d (8.5)	141.2	7.52 d (8.6)	141.0	7.49 d (8.5)
4	107.3	6.64 d (8.5)	107.3	6.63 d (8.6)	107.1	6.61 d (8.5)
4a	158.4		158.4		158.8	
5	82.7	4.81 d (6.9)	82.7	4.81 d (6.8)	76.1	4.05 d (6.7)
6	33.5	2.99 *p* (7.7)	33.3	2.98 *p* (7.4)	30.3	2.38 m
7	36.5	2.71 dd (17.3, 8.3)	36.5	2.71 dd (17.3, 8.3)	39.6	2.60 q (9.1)
		2.49 dd (17.3, 8.0)		2.48 dd (17.3, 7.9)		2.39 dd (9.1, 5.9)
8	174.7		174.7		173.0	
8a	39.6	3.28 d (17.3) (17.3)	39.6	3.28 d (17.0) (17.0)	40.0	3.26 brs
		3.21 d		3.21 d		
9	194.1		194.1		195.8	
9a	107.1		107.1		107.5	
10a	84.3		83.9		86.9	
11	14.8	1.34 d (7.2)	14.8	1.34 d (7.2)	13.5	1.07 d (6.4)
12	169.1		169.1		170.4	
13	53.6	3.78 s	53.6	3.77 s	53.1	3.76 s
14					51.6	3.70 s
1-OH		11.92 s		11.91 s		12.01 s
5-OH						2.76 d (7.2)
1′	159.7		159.2		159.2	
2′	117.4		117.4		117.4	
3′	141.4	7.53 d (8.5)	141.2	7.52 d (8.6)	141.4	7.52 d (8.6)
4′	107.3	6.64 d (8.5)	107.3	6.63 d (8.6)	107.1	6.62 d (8.6)
4a′	158.4		158.4		158.8	
5′	82.7	4.81 d (6.9)	87.5	4.46 d (4.0)	87.4	4.46 d (3.9)
6′	33.5	2.99 p (7.7)	29.9	2.84 m	29.6	2.84 m
7′	36.5	2.71 dd (17.3, 8.3)	35.9	2.92 dd (17.8, 9.4)	35.8	2.92 dd (17.8, 9.3)
		2.49 dd (17.3, 8.0) 2.49 dd (17.3, 8.0)		2.24 dd (17.8, 4.6) 2.24 dd (17.8, 4.6)		2.24 dd (17.8, 4.5) 2.24 dd (17.8, 4.5)
8′	174.7		175.2		174.7	
8a′	39.6	3.28 d (17.3)	39.5	3.22 d (17.0)	39.3	3.21 d (17.0)
		3.21 d (17.3)		3.06 d (17.0)		3.06 d (17.0)
9′	194.1		194.1		194.1	
9a′	107.1		107.1		107.5	
10a′	84.3		83.9		84.3	
11′	14.8	1.34 d (7.2)	20.7	1.29 d (6.9)	20.6	1.29 d (6.9)
12′	169.1		168.7		168.7	
13′	53.6	3.78 s	53.6	3.77 s	53.5	3.77 s
1′-OH OH′		11.92 s				
5′-OH OH′				11.91 s		12.02 s

*Aculeaxanthone C (**6**)*: yellow powder; ${\left[\alpha \right]}_{D}^{20}$ −5 (*c* 0.067, MeOH); ECD (1.7 × 10^−4^ M, MeOH), *λ* [nm] (Δ*ε*) 292 (+1.01), 246 (−0.36), 233 (+0.75), 213 (−0.83); ^1^H and ^13^C NMR (CDCl_3_), see Table 2; HR-ESIMS (*m/z*): 661.1526 [M + Na]^+^ (calcd for C_32_H_30_O_14_Na, 661.1533).

*Aculeaxanthone D (**7**)*: yellow powder; ${\left[\alpha \right]}_{D}^{20}$ +5 (*c* 0.02, MeOH); ECD (1.6 × 10^−4^ M, MeOH), *λ* [nm] (Δ*ε*) 339 (+0.16), 244 (+0.47), 214 (−0.69); ^1^H and ^13^C NMR (CDCl_3_), see Table 2; HR-ESIMS (*m/z*): 693.1789 [M + Na]^+^ (calcd for C_33_H_34_O_15_Na, 693.1795).

*Aculeaxanthone E (**8**)*: yellow powder; ${\left[\alpha \right]}_{D}^{20}$ +28.5 (*c* 0.067, MeOH); ECD (1.7 × 10^−4^ M, MeOH), *λ* [nm] (Δ*ε*) 379 (+6.18), 338 (−0.66), 295 (+0.03), 244 (−6.65), 220 (+0.81); ^1^H and ^13^C NMR (CDCl_3_), see Table 1; HR-ESIMS (*m/z*): 677.1472 [M + Na]^+^ (calcd for C_32_H_30_O_15_Na, 677.1482).

### Computational analysis

The conformational majorization of the stereoisomers was achieved using computational TDDFT calculations. To perform the conformational analysis, MMFF94 molecular mechanics was carried out. The ground-state geometries of those stereoisomers were further optimized using Gaussian 09 (Frisch et al., 2009). Vibrational evaluation was finished using TDDFT calculations at the B3LYP/6-311G (2d, p) level to determine minima. The Boltzmann distribution law (Eq. 1) was used to calculate the equilibrium populations at room-temperature. The overall theoretical ECD spectra were simulated with a Gaussian function and then acquired according to the Boltzmann weighting.


(1) $$\frac{N_{i}}{N}=\frac{g_{i}e^{-\frac{E_{i}}{k_{B}T}}}{\sum g_{i}e^{-\frac{E_{i}}{k_{B}T}}}$$

In this case, *N_i_* represents the number of conformers *i* with degeneracy *g_i_* and energy *E_i_* at temperature *T*, and *k*_B_ is Boltzmann constant.

### Antibacterial assay

All the isolates were tested against Gram-positive bacteria including *Enterococcus faecium* ATCC 19434, *Bacillus subtilis* 168, *Staphylococcus aureus* ATCC 25923 and MRSA USA300, Gram-negative bacteria including *Helicobacter pylori* 129, G27, as well as 26695, and multi drug-resistant strain *H. pylori* 159, and one Mycobacterium *M. smegmatis* ATCC 607. The MIC of anaerobic bacteria was examined by a broth microdilution assay. Briefly, twofold serial dilutions of compounds **1**–**9** were prepared in 96-well microtiter plates. *H. pylori* liquid cultures was also diluted with BHI broth and was inoculated into each well to get a final concentration of 5 × 10^5^ CFU/ml. After incubation in a microaerophilic atmosphere at 37°C for 72 h, the MIC was confirmed to be the lowest concentration which resulted in no turbidity. Metronidazole was used as a positive control. To obtain MICs for aerobic bacteria, the agar dilution streak method recommended in Clinical and Laboratory Standards Institute document (CLSI) M07-A10 was used. The broth was diluted with saline and applied to plates, delivering a final concentration of approximately 10^5^ CFU/spot.

### Cytotoxicity assay

Compounds **1**–**9** was tested against the Bel-7402, A-549 and HCT-116 cell lines according to MTT assay. All the isolates were dissovled and diluted using dimethyl sulfoxide (DMSO). Cells were seeded at 4000 cells in 96-well microplates and incubated for 24 h and spent with the isolates for 72 h. After that, each well was treat for 4 h with MTT reagent. By operating a microplate reader, absorbance at 570 nm was measured after replacing the medium with 100 μl of DMSO. All compounds were tested three times independently (*n* = 3). 5-Fluorouracil was applied to positive control. Finally, the Logit method was applied to caculate IC_50_ values.

## Results and discussion

Compounds **1**–**6** and **9** gave the same molecular formula of C_32_H_30_O_14_, deduced by HR-ESIMS, providing 18 unsaturation degrees. Compounds **1**, **2**, **3** and **9** were determined to be secalonic acid D, chrysoxanthones B, C, and 4–4′-secalonic acid D, respectively, by detailed comparison of their specific rotation values and NMR data with literatures (El-Elimat et al., 2015; Qin et al., 2015b; Zhen et al., 2018). The absolute configurations of **2**, **3** and **9** were also confirmed by TDDFT-ECD calculation (Supplementary Figures S5, S9, S52).

**Figure 2:**
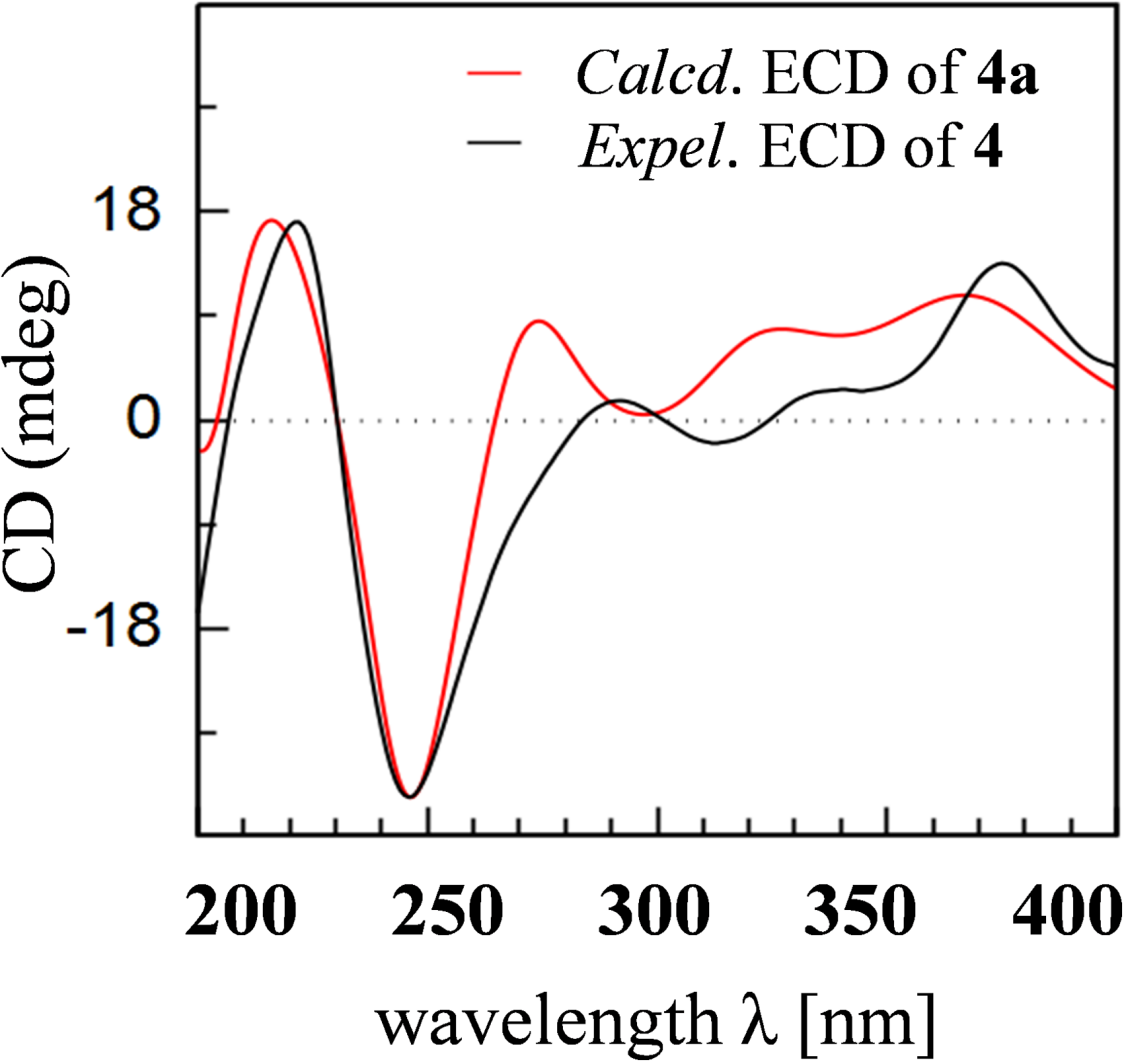
Experimental ECD spectrum of **4** and calculated ECD spectrum of **4a**.

Aculeaxanthone A (**4**) was obtained as an orange powder. Compound **4** provided the near-identical NMR data to those of **2**. The significant difference appeared in **4** was the HMBC correlations from the H-2 and hydroxyl proton 1-OH to C-9a, which suggested the C-4–C-2′ linkage for **4** instead of C-2–C-2′ linkage in **2**. The tetrahydroxanthone and the chromanone monomers in **4** connected with a C-4–C-2′ linkage was determined by the COSY correlations of H-2/H-3 instead of H-4/H-3, and HMBC correlations of H-3 with C-2′ and C-4a and H-3′ with C-4. The relative configuration of the two monomers in **4** was the same to those in **2**, as indicated by the coupling constants (Table 1) and the interpretation of the NOE signals, together with the biogenetic consideration. To confirm the absolute configuration of **4**, the calculated ECD spectrum of **4a** were acquired according to the TDDFT calculations (Grkovic et al., 2007; Bringmann et al., 2009). The Molecular Operating Environment (MOE) was performed to conduct the systematic conformational analysis for **4a** (5*R*, 6*S*, 10a*R*, 5’*R*, 6’*R*, 10a’*R*) according to the Merck Molecular Force Field (MMFF). The lowest energy conformers were obtained after we reoptimize the designated stereoisomer according to TDDFT at the B3LYP/6-311G (2d, p) (Grimme, 2006) level. These were further filtered to gain the principal conformer on the base of the Boltzmann distribution. Finally, Gaussian broadening was used to provide the complete calculated ECD spectrum of **4a**. Obviously, the experimental and calculated ECD spectra for **4** was in great agreement (Figure 2), indicating that an 5*R*, 6*S*, 10a*R*, 5’*R*, 6’*R*, 10a’*R* absolute configuration could be assigned to **4**.

**Figure 3:**
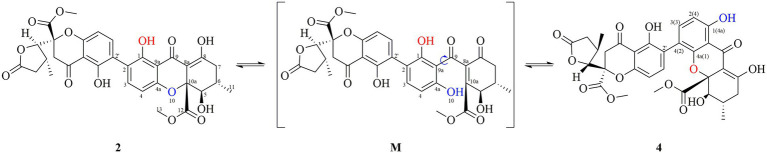
Proposed interconversion mechanism between **2** and **4**.

Compound **4** was found unstable in DMSO-*d*_6_, consistent with the findings of Wu et al. (2015). Then the central chirality elements of **2** and **4** was assigned by chemical conversions. The conversion was monitored by ^1^H-NMR spectra and the product was isolated using a shimodzu HPLC system. The Wessely-Moser rearrangement between **2** (2′–2 linkage) and **4** (2′–4 linkage) was represented in Figure 3, which further confirmed the absolute configuration of **4**.

**Figure 4:**
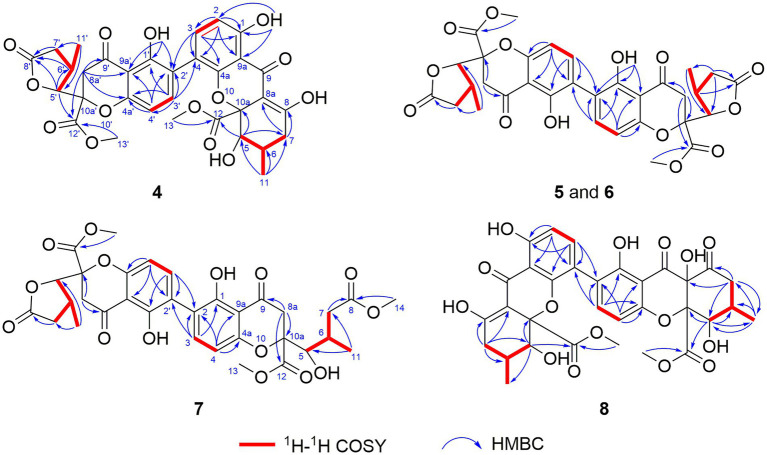
^1^H-^1^H COSY and HMBC correlations of **4**–**8**.

**Figure 5:**
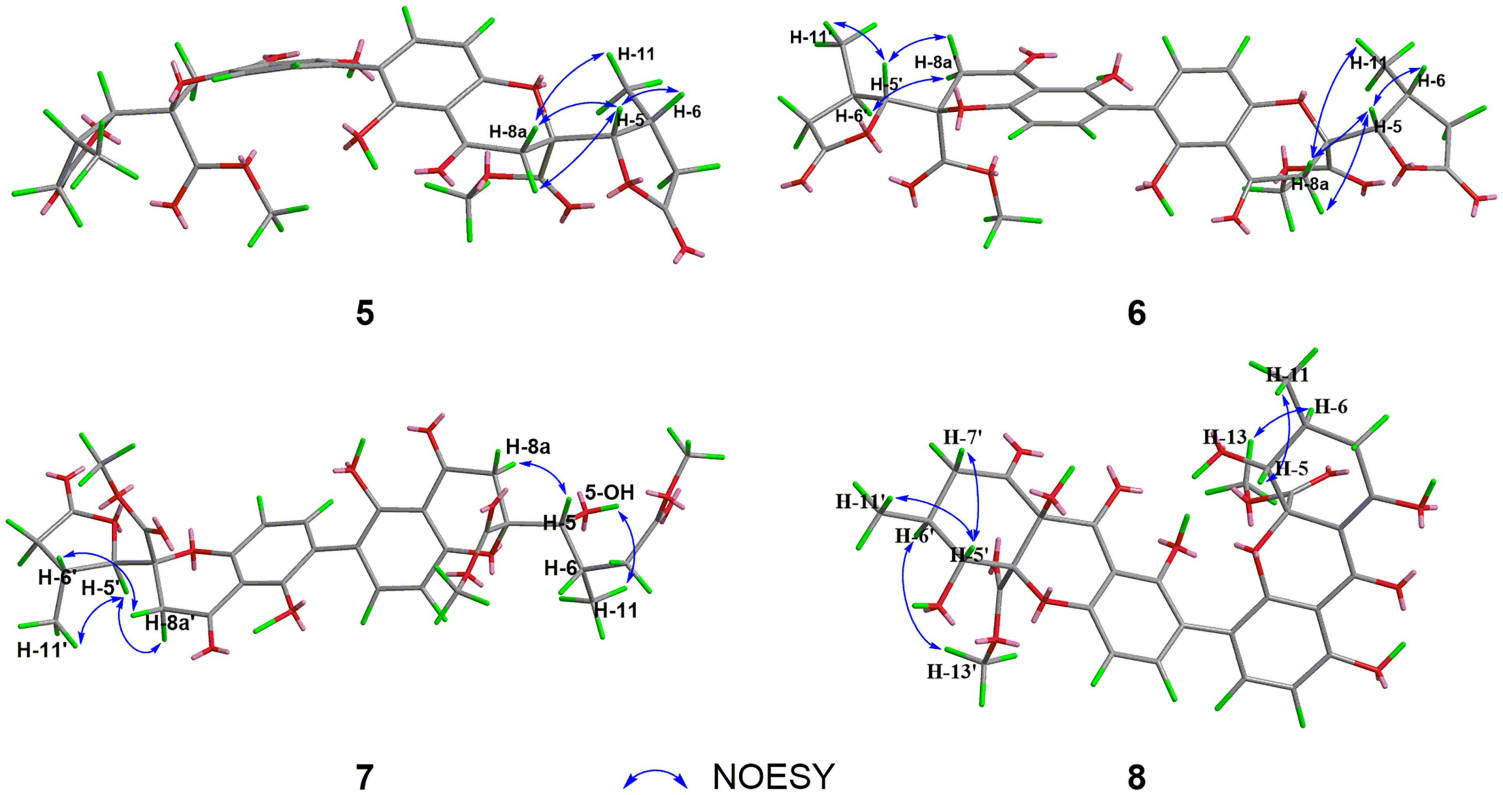
NOESY correlations of **5**–**8**.

**Figure 6:**
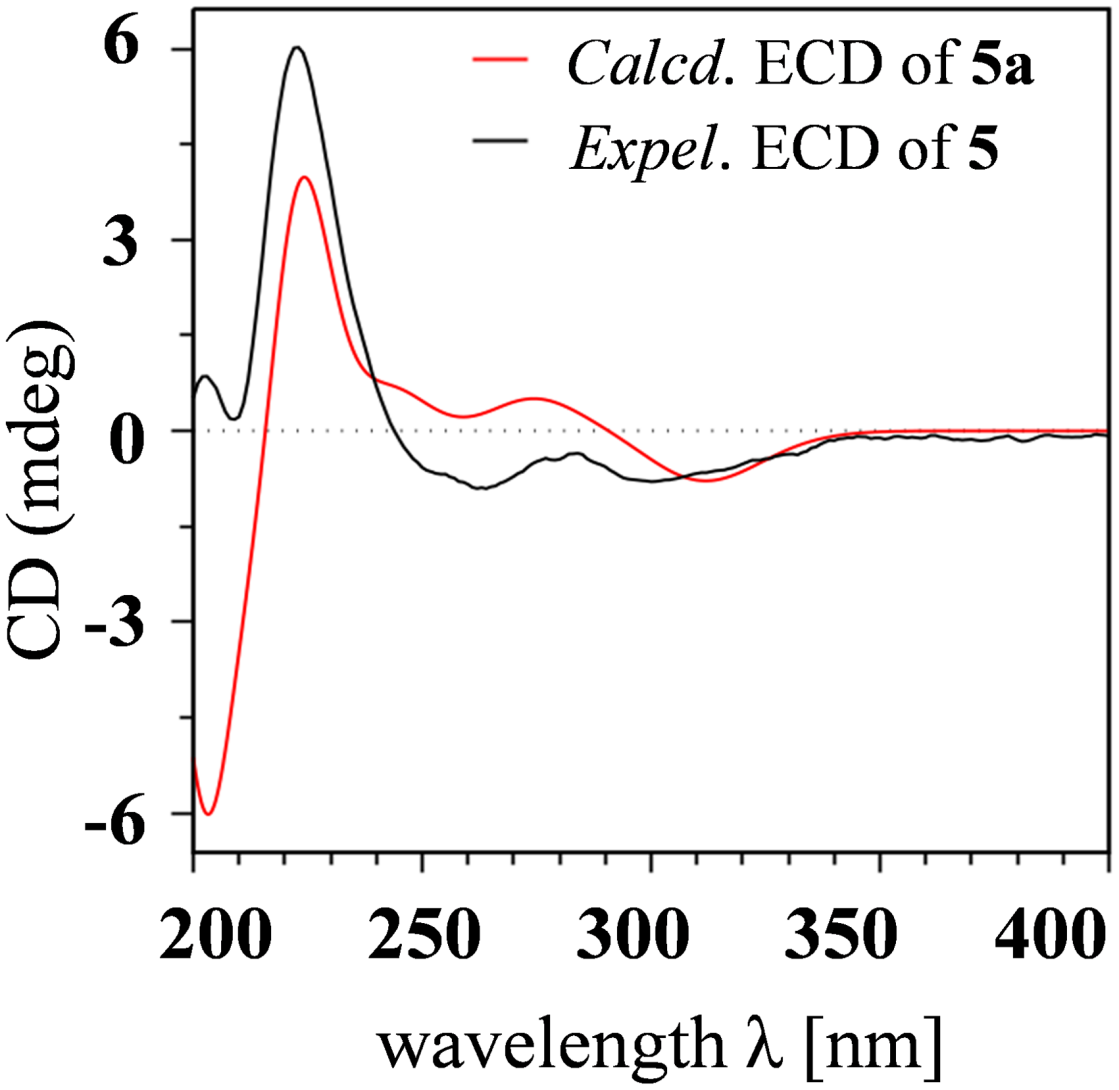
Experimental ECD spectrum of **5** and calculated ECD spectrum of **5a**.

Aculeaxanthone B (**5**) was acquired as a yellow powder. Its 1D NMR data displayed only half as many carbon resonances as expected, which were assigned as three carbonyls (*δ*_C_ 174.7, 194.1, and 169.1), six aromatic carbons (*δ*_C_ 159.7, 117.4, 141.4, 158.4, and 107.1), one nonprotonated sp^3^ carbon linked to oxygen (*δ*_C_ 84.3), two methines (*δ*_C_ 82.7 and 33.5), two methylenes (*δ*_C_ 36.5 and 39.6), one methyl (*δ*_C_ 14.8), and one methoxyl (*δ*_C_ 53.6). Analysis of ^1^H NMR spectrum indicated one aromatic ring, two methylenes, one oxymethine, one methine, one methyl, and one methoxyl (Table 2). These evidence indicated that **5** must be a symmetric homodimer of two chromanone lactone monomers. The HMBC correlations of H-5 with C-10a and C-12 determined the connection between the lactone moiety and the chromanone monomeric unit (Figure 4). The 2–2′ linkage of **5** was established by the HMBC correlations of 1-OH with C-2 and C-9a, H-3 (H-3′) with C-1 (C-1′), C-4a (C-4a’), and C-2′ (C-2) (Figure 4). The NOESY spectrum was used to provide the relative configuration of **4** (Figure 5). The strong NOESY correlations from H-5 (H-5′) to H-6 (H-6′) and H_α_-8a (H_α_-8a’) suggested that these protons provided the co-facial orientation, which was also determined by the evidence of the coupling constant (^3^*J*_H-5,H-6_ = 6.9 Hz) with analogues in literatures (Zhang et al., 2008; El-Elimat et al., 2015; Wu et al., 2015). For 5*S*, 6*R*, 10a*R*, 5’*S*, 6’*R*, 10a’*R*, the experimental spectrum agreed well with the calculated one, which unequivocally assigns the absolute configuration of **5** (Figure 6).

**Figure 7:**
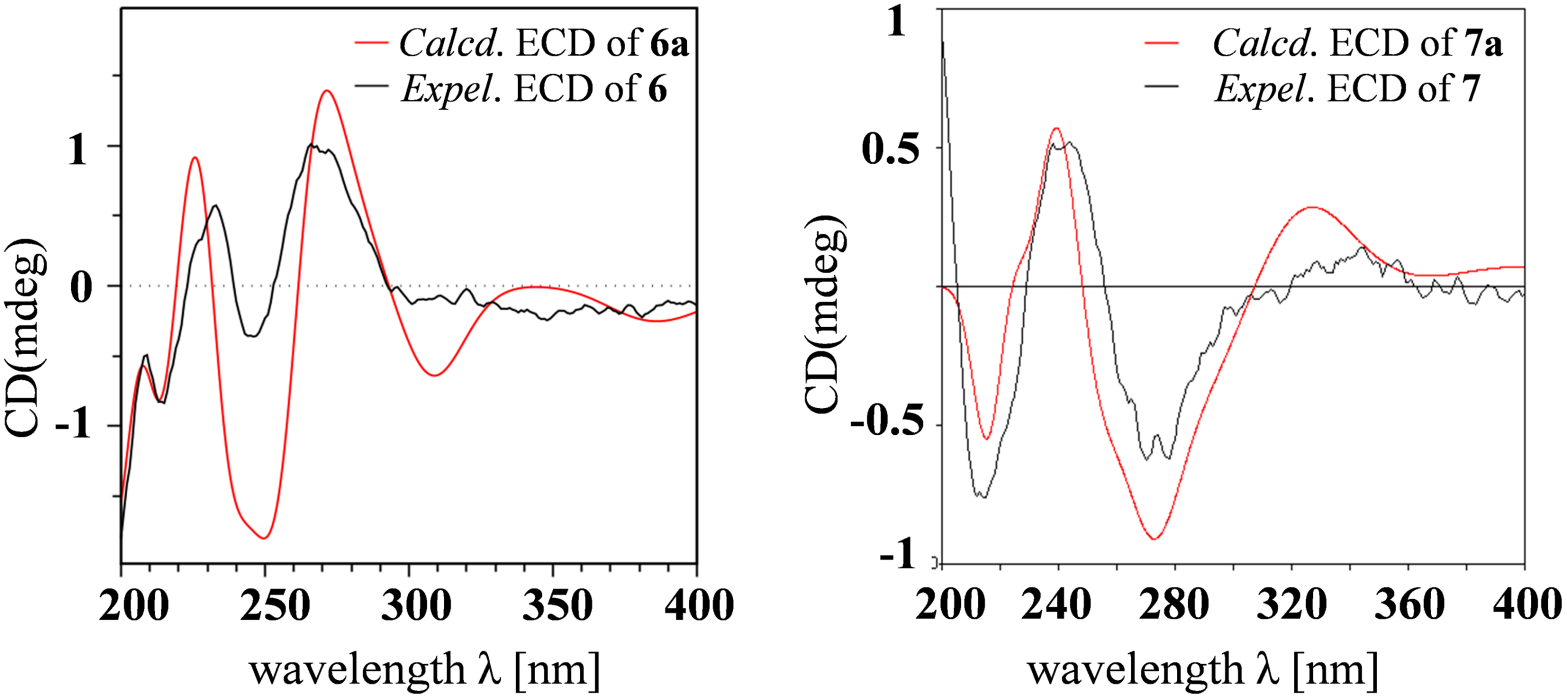
Experimental ECD spectra of **6** and **7** and calculated ECD spectra of **6a** and **7a**.

Aculeaxanthone C (**6**) was also found to possess the identical molecular formula (C_32_H_30_O_14_) to **2**–**5**, as suggested by the HR-ESIMS ions observed at *m/z* 661.1526 (calcd for C_32_H_30_O_14_Na^+^, 661.1533). A scrupulous analysis of the 1D NMR data of **6** and **5** (Table 2) indicated **6** to be a heterodimer of two different chromanone lactone monomers. Subtraction of the signals of aculeaxanthone B (**5**) subunit confirmed the near-identical remaining NMR data with those of the chromanone lactone of **2**. The relative configuration of one chromanone lactone in **6** was determined by the NOESY correlation of H-5/H-6 (Figure 5). The configuration of H-5′ and H-6′ in another chromanone lactone monomer was determined as the same as that of **2**, confirmed by the NOESY correlation of H-5′ with H-11′ (Figure 5). To establish the absolute configuration of **6**, the lowest energy conformer was calculated. Distinctly, the experimental ECD spectrum for **6** and the calculated one for **6a** can be found a great fit (Figure 7). Finally, the 5*S*, 6*R*, 10a*R*, 5’*R*, 6’*R*, 10a’*R* configuration could be assigned to **6** (Figure 1).

Aculeaxanthone D (**7**) was derived as a yellow powder. Its molecular formula was deduced as C_33_H_34_O_15_ from the HR-ESIMS ions at *m/z* [M + Na]^+^ 693.1789, indicating that **7** presented one more carbon and one less unsaturation degree than compounds **1**–**6**. The 1D NMR data (Table 2) displayed that the chromanone lactone monomer of **7** was identical to that of **2** and **6**. Compound **7** was determined to possess 2–2′ linkage by the HMBC correlations from H-3 (*δ*_H_ 7.49) to C-2′ (*δ*_C_ 117.4) and H-3′ (*δ*_H_ 7.52) to C-2 (*δ*_C_ 117.4). The distinction difference was that a side chain in **7** replaced the cyclohexene moiety in **2**, determined by the HMBC correlations of the methoxyl H_3_-14 (*δ*_H_ 3.70) with C-8 (*δ*_C_173.0). The relative configurations of the chromanone monomer in **7** were identical to those in **2** and **6** from the NOESY correlations between H-5′ with H-11′(Figure 5), the chemical shifts, and biogenetic grounds. The anti relationship between H-5 with H-6 in **7** were proposed to be the same as those in **2**, deduced from the coupling constants (^3^*J*_H-5,H-6_ = 6.7 Hz) and biogenetic consideration. To gain the absolute configuration of **7**, the ECD spectrum for the lowest energy conform **7a** (5*R*, 6*S*, 10a*R*, 5’*R*, 6’*R*, 10a’*R*), was calculated and compared with the experimental one. Notably, the calculated ECD spectrum showed good fitting with the experimental one (Figure 7), determining that an 5*R*, 6*S*, 10a*R*, 5’*R*, 6’*R*, 10a’*R* absolute configuration could be defined to **7**.

**Figure 8:**
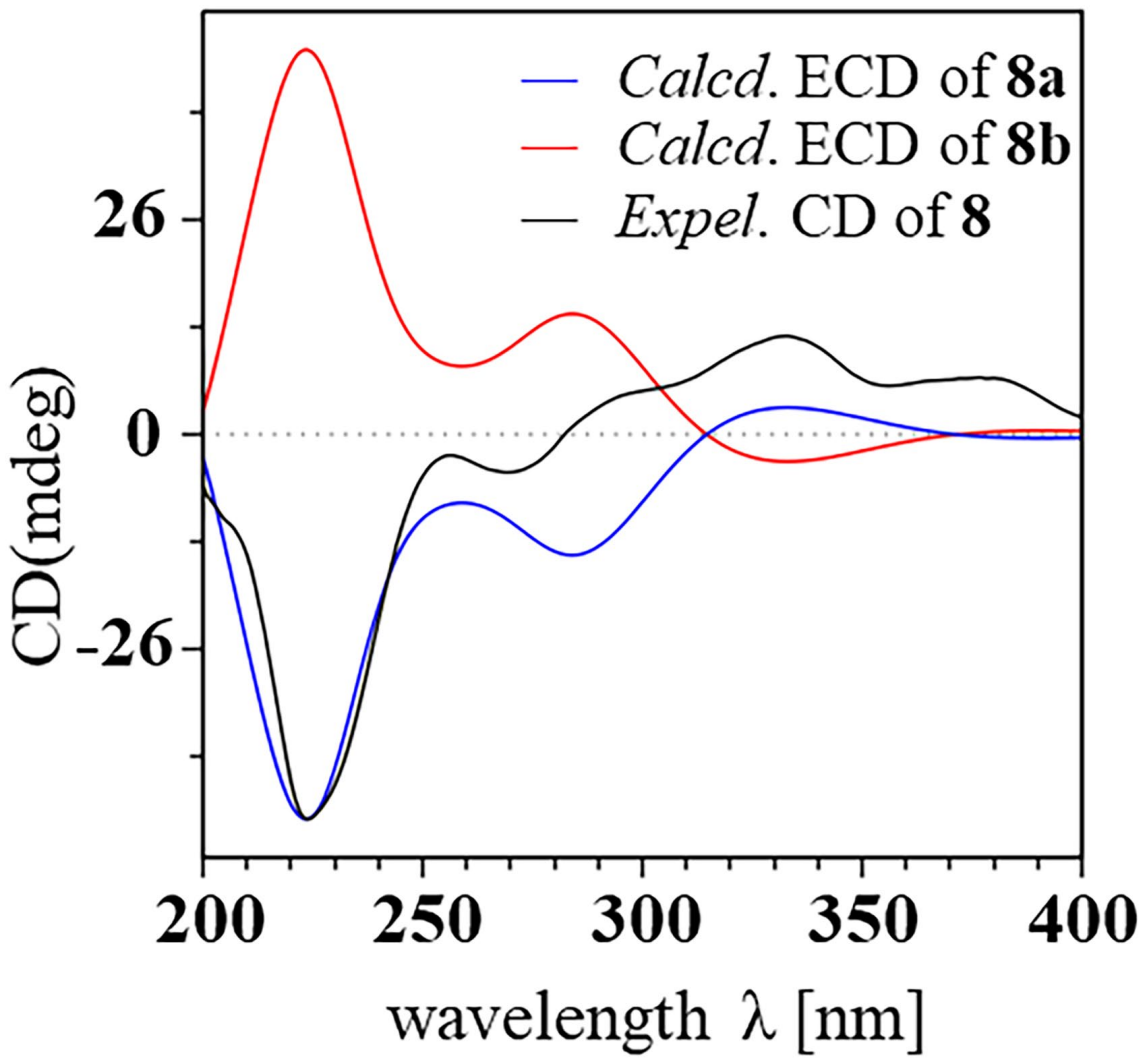
Experimental and calculated ECD spectra of compound **8**.

The molecular formula of aculeaxanthone E (**8**) was assigned as C_32_H_30_O_15_, 16 mass units greater than that of compounds **1**–**6**, according to its HR-ESIMS ion at *m/z* 677.1472 [M + Na]^+^ (calcd for 677.1482). The ^13^C NMR and HSQC spectra (Table 1) showed 32 signals corresponding to five carbonyls (*δ*_C_ 187.1, 170.3, 198.5, 191.6, and 167.9), two 1,2,3,4-substituted benzene rings, one double band (*δ*_C_ 177.7 and 101.5), three nonprotonated sp^3^ carbons linked to oxygen (*δ*_C_ 84.8, 71.8, and 89.4), two oxymethines (*δ*_C_ 76.8 and 74.0), two methylenes (*δ*_C_ 36.3 and 43.4), two methoxyls (*δ*_C_ 53.3 and 53.6), two methines (*δ*_C_ 29.2 and 32.0), and two methyls (*δ*_C_ 18.0 and 18.5) (Table 1). Inspection of the 1D NMR data of **8** with those in earlier reported (Wang et al., 2018) suggested **8** to be a heterodimer of tetrahydroxanthone monomer and hexahydroxanthone monomer. Careful analysis of the 2D NMR spectra suggested that the tetrahydroxanthone monomer of **8** was identical to those of compounds **1**–**4**. Subtraction of the signals of the tetrahydroxanthone monomer indicated the similitube of the remaining NMR data with those of penibishexahydroxanthone A (Chen M. et al., 2019). The 2–4′ linkage was determined by the HMBC correlations of H-3 with C-2′ and H-3′ with C-4 (Figure 4). Then, the planar structure of **8** as displayed in Figure 1. The tetrahydroxanthone monomer in **8** was easily determined to be identical to those of compounds **1**–**4** by the NOESY correlation from H-5 to H_3_-11 (Figure 5), the large ^3^*J*_H-5, H-6_ (11.2 Hz) value. The large ^3^*J*_H-5’,H-6′_ (10.7 Hz) value, strong NOESY correlation of H-5′ with H_3_-11′, and the near-identical chemical shifts with those of penibishexahydroxanthone A suggested that the hexahydroxanthone monomer was identical to penibishexahydroxanthone A. To determine the absolute configuration of **8,** TDDFT-ECD spectra of **8a** (5*R*, 6*S*, 10a*S*, 5’*R*, 6’*S*, 8a’*R*, 10a’*S*) and **8b** (5*S*, 6*R*, 10a*R*, 5’*S*, 6’*R*, 8a’*S*, 10a’*R*) were calculated (Figure 8). After that, **8** was assigned as 5*R*, 6*S*, 10a*S*, 5’*R*, 6’*S*, 8a’*R*, 10a’*S*, as shown in Figure 1.

Compound **9** possessed the same molecular formula (C_32_H_30_O_14_) as secalonic acid D (**1**) which was based on its HR-ESIMS analysis (*m/z* 661.1530; [M + Na]^+^, calcd for 661.1533), suggesting it to be an isomer of **1**. Similar to **5**, **9** displayed half of the expected carbon signals, suggesting a structurally symmetrical. Careful analysis of the NMR spectra, **9** displayed identical 1D NMR data to those of 4–4′-secalonic acid D, 4–4′-secalonic acid A (Chen L. et al., 2019), and talaroxanthone (Koolen et al., 2013), suggesting that **9** shared the same planar structure and relative configurations as those compounds. Compound **9** was finally determined to be 4–4′-secalonic acid D by comparison their specific rotation values, of which the absolute configuration was further determined by the TDDFT-ECD calculation (Figure S52). However, after comparison of the 1 D NMR data of talaroxanthone with those of 4–4′-secalonic acid A, the structure of talaroxanthone should be revised to 4–4′-secalonic acid A (Koolen et al., 2013), due to their identical NMR data, particularly the large ^3^*J*_H-5, H-6_ (12.0 Hz) value, and the specific rotation values (Koolen et al., 2013; Qin and Porco, 2015).

Compounds **1**–**9** were tested for cytotoxicity against the Bel-7402, A549, and HCT-116 cancer cell lines. Of the dimeric tetrahydroxanthone derivatives, only compound **6** provided cytotoxicity effect against Bel-7402 cell line (IC_50_, 1.96 μM). Additionally, antimicrobial activity was evaluated for all dimeric tetrahydroxanthones, including four Gram-positive bacteria including *Enterococcus faecium* ATCC 19434, *B. subtilis* 168, *S. aureus* ATCC 25923 and MRSA USA300; four Gram-negative bacteria, including *H. pylori* 129, G27, as well as 26695, and multi drug-resistant strain *H. pylori* 159, and one Mycobacterium *M. smegmatis* ATCC 607. However, only compound **1** performed activities against *H. pylori* G27, *H. pylori* 26695, *H. pylori* 129, *H. pylori* 159, *S. aureus* USA300, and *B. subtilis* 168 with MIC values of 4.0, 4.0, 2.0, 2.0, 2.0 and 1.0 μg/ml, respectively.

## Conclusion

In summary, five new dimeric tetrahydroxanthones (**4**–**8**) with a high degree of structural complexity and diversity were separated from the culture of the marine-derive fungus *A. aculeatinus* WHUF0198. Compound **4** represented the chemical conversion product of **2**, indicating the possibility that some dimeric tetrahydroxanthones might produce spontaneously from the natural dimers in the process of the fermentation or extraction. Compounds **5** and **6** contained two chromanone monomers coupled by a 2–2′ linkage to form a symmetric homodimer and an asymmetric dimer, respectively. Compound **7** included a common chromanone lactone unit and a ring-opened tetrahydroxanthone monomer, which might derive from **2** instead of the methanolysis product of **6**. Thus, compound **8** represented the fifth dimeric hexahydroxanthones, of which the common tetrahydroxanthone monomer and the hexahydroxanthone monomer were connected by a 2–4′ linkage. Furthermore, the structure of talaroxanthone should be revised to 4–4′-secalonic acid A based on their identical NMR data and specific rotation values. The absolute configurations of all dimeric tetrahydroxanthones were determined by a combination of ECD calculation, chemical conversions, specific rotations, and biogenetic consideration. Compound **6** showed cytotoxicity effect against Bel-7402 cell line with an IC_50_ value of 1.96 μM, and compound **1** provided activities against *H. pylori* G27, *H. pylori* 26695, *H. pylori* 129, *H. pylori* 159, *S. aureus* USA300, and *B. subtilis* 168 with MIC values of 4.0, 4.0, 2.0, 2.0, 2.0 and 1.0 μg/ml, respectively.

## Data availability statement

The original contributions presented in the study are included in the article/Supplementary material, further inquiries can be directed to the corresponding authors.

## Author contributions

Y-SC, KH, and HB: conceptualization, methodology, and writing—review and editing. JW, HS, and K-KZ: data curation. Y-SC, HS, and MeZ: funding acquisition. K-KZ: software. JW, YZ, and HS: chemical investigation. MiZ, S-BW, and HB: bioactivity assays. KH: fungal resources. JW, HS, and Y-SC: data analysis. JW, MeZ, and HS: writing—original draft preparation. All authors contributed to the article and approved the submitted version.

## Funding

This research was supported by the National Key Research and Development Program of China (no. 2021YFC2100600), the National Natural Science Foundation of China (nos. 81973201 and 82204225), the Natural Science Foundation of Hubei Province (nos. 2021CFB347 and 2021CFB061), and the Joint Fund of Health Commission of Hubei Province (no. WJ2019H024).

## Conflict of interest

The authors declare that the research was conducted in the absence of any commercial or financial relationships that could be construed as a potential conflict of interest.

The handling editor DZ declared a shared parent affiliation with the author S-BW at the time of review.

## Publisher’s note

All claims expressed in this article are solely those of the authors and do not necessarily represent those of their affiliated organizations, or those of the publisher, the editors and the reviewers. Any product that may be evaluated in this article, or claim that may be made by its manufacturer, is not guaranteed or endorsed by the publisher.

## References

[ref1] Bringmann G. Bruhn T. Maksimenka K. Hemberger Y. (2009). The assignment of absolute stereostructures through quantum chemical circular dichroism calculations. Eur. J. Org. Chem. 2009, 2717–2727. doi: 10.1002/ejoc.200801121

[ref2] Cai S. King J. B. Du L. Powell D. R. (2014). Cichewicz, R H. bioactive sulfur-containing sulochrin dimers and other metabolites from an *Alternaria* sp. isolate from a Hawaiian soil sample. J. Nat. Prod. 77, 2280–2287. doi: 10.1021/np5005449, PMID: 25265160 PMC4208674

[ref3] Cai Y.-S. Sarotti A. M. Zhou T.-L. Huang R. Qiu G. Tian C.. (2018). Flabellipparicine, a flabelliformide-apparicine-type bisindole alkaloid from *Tabernaemontana divaricata*. J. Nat. Prod. 81, 1976–1983. doi: 10.1021/acs.jnatprod.8b00191, PMID: 30169038

[ref4] Cao H.-Y. Yi C. Sun S.-F. Li Y. Liu Y.-B. (2022). Anti-inflammatory dimeric tetrahydroxanthones from an endophytic *Muyocopron laterale*. J. Nat. Prod. 85, 148–161. doi: 10.1021/acs.jnatprod.1c00878, PMID: 35029398

[ref5] Chen M. Gui Y. Zhu H. Zhang Z. Lin H.-W. (2019). Proangiogenic penibishexahydroxanthone a from the marine-derived fungus *Penicillium* sp. ZZ486A. Tetrahedron Lett. 60, 1393–1396. doi: 10.1016/j.phytochem.2018.04.021

[ref6] Chen L. Li Y.-P. Li X.-X. Lu Z.-H. Zheng Q.-H. Liu Q.-Y. (2019). Isolation of 4,4′-bond secalonic acid D from the marine-derived fungus *Penicillium oxalicum* with inhibitory property against hepatocellular carcinoma. J. Antibiot. 72, 34–44. doi: 10.1038/s41429-018-0104-5, PMID: 30258223

[ref7] Deshmukh S. K. Mishra P. D. Kulkarni-Almeida A. Verekar S. Sahoo M. R. Periyasamy G.. (2009). Anti-inflammatory and anticancer activity of ergoflavin isolated from an endophytic fungus. Chem. Biodivers. 6, 784–789. doi: 10.1002/cbdv.200800103, PMID: 19479845

[ref8] El-Elimat T. Figueroa M. Raja H. A. Graf T. N. Swanson S. M. Falkinham J. O. III. (2015). Biosynthetically distinct cytotoxic polyketides from *Setophoma terrestris*. Eur. J. Org. Chem. 2015, 109–121. doi: 10.1002/ejoc.201402984, PMID: 25574154 PMC4283843

[ref9] Franck B. (1969). Structure and biosynthesis of the ergot pigments. Angew. Chem. Int. Edit. 8, 251–260. doi: 10.1002/anie.196902511, PMID: 4977570

[ref10] Franck B. (1980). “The biosynthesis of the ergochromes” in The Biosynthesis of Mycotoxins: A Study in Secondary Metabolism. ed. Steyn P. S. (New York: Academic Press), 157–191.

[ref11] Frisch M. J. Trucks G. W. Schlegel H. B. Scuseria G. E. Robb M. A. Cheeseman J. R.. (2009). Investigation of Structural and Electronic Properties of [Tris (Benzene-1,2-Dithiolato) M] 3- (M = V, Cr, Mn, Fe and Co) Complexes: A Spectroscopic and Density Functional Theoretical Study. Gaussian 09, Revision A.02, Gaussian, Inc., Wallingford, CT.

[ref12] Grimme S. (2006). Semiempirical GGA-type density functional constructed with a long-range dispersion correction. J. Comput. Chem. 27, 1787–1799. doi: 10.1002/jcc.20495, PMID: 16955487

[ref13] Grkovic T. Ding Y. Li X. C. Ferreira D. (2007). Theoretical calculation of electronic circular dichroism of the rotationally restricted 3, 8′ -biflavonoid morelloflavone. J. Org. Chem. 72, 9010–9017. doi: 10.1021/jo801622n, PMID: 17958369

[ref14] Koolen H. H. F. Menezes L. S. Souza M. P. Silva F. M. A. Almeida F. G. O. de Souza A. Q. L.. (2013). Talaroxanthone, a novel xanthone dimer from the endophytic fungus *Talaromyces* sp. associated with duguetia stelechantha (Diels) R. E. Fries. J. Brazil. Chem. Soc. 24, 880–883. doi: 10.5935/0103-5053.20130104

[ref15] Lombe B. K. Feineis D. Bringmann G. (2019). Dimeric naphthylisoquinoline alkaloids: polyketide-derived axially chiral bioactive quateraryls. Nat. Prod. Rep. 36, 1513–1545. doi: 10.1039/C9NP00024K, PMID: 31134266

[ref16] Luenne F. Koehler J. Stroh C. Mueller L. Daniliuc C. G. Mueck-Lichtenfeld C.. (2021). Insights into ergochromes of the plant pathogen *Claviceps purpurea*. J. Nat. Prod. 84, 2630–2643. doi: 10.1021/acssuschemeng.8b00102, PMID: 34553942

[ref17] Lv X. J. Ding F. Wei Y. J. Tan R. X. (2021). Antiosteoporotic tetrahydroxanthone dimers from aspergillus brunneoviolaceus FB -2 residing in human gut. Chin. J. Chem. 39, 1580–1586. doi: 10.1002/cjoc.202100026

[ref18] Masters K.-S. Brase S. (2012). Xanthones from fungi, lichens, and bacteria: the natural products and their synthesis. Chem. Rev. 112, 3717–3776. doi: 10.1021/cr100446h, PMID: 22617028

[ref19] Phang Y. L. Zheng C. Xu H. (2022). Structural diversity and biological activities of caged Garcinia xanthones: recent updates. Acta Mater. Med. 22, 72–95. doi: 10.3390/molecules22122026, PMID: 29168781 PMC6149763

[ref20] Qin T. Iwata T. Ransom T. T. Beutler J. A. Porco J. A. (2015a). Syntheses of dimeric tetrahydroxanthones with varied linkages: investigation of "shapeshifting" properties. J. Am. Chem. Soc. 137, 15225–15233. doi: 10.1021/jacs.5b09825, PMID: 26544765 PMC4863954

[ref21] Qin T. Porco J. A. (2015). Total syntheses of secalonic acids a and D. Angew. Chem. Int. Edit. 53, 3107–3110. doi: 10.1002/anie.201311260, PMID: 24519991 PMC4098722

[ref22] Qin T. Skraba-Joiner S. L. Khalil Z. G. Johnson R. P. Capon R. J. Porco J. A. (2015b). Atropselective syntheses of (−) and (+) rugulotrosin a utilizing point-to-axial chirality transfer. Nat. Chem. 7, 234–240. doi: 10.1038/nchem.2173, PMID: 25698333 PMC4339264

[ref23] Rezanka T. Sigler K. (2007). Hirtusneanoside, an unsymmetrical dimeric tetrahydroxanthone from the lichen *Usnea hirta*. J. Nat. Prod. 70, 1487–1491. doi: 10.1021/np070079m, PMID: 17822296

[ref24] Roensberg D. Debbab A. Mandi A. Vasylyeva V. Boehler P. Stork B.. (2013). Pro-apoptotic and immunostimulatory tetrahydroxanthonedimers from the endophytic fungus *Phomopsis longicolla*. J. Org. Chem. 78, 12409–12425. doi: 10.1016/j.tetlet.2015.03.126, PMID: 24295452

[ref25] Sadorn K. Saepua S. Boonyuen N. Choowong W. Rachtawee P. Pittayakhajonwut P. (2021). Bioactive dimeric tetrahydroxanthones with 2,2′- and 4,4′-axial linkages from the entomopathogenic fungus *Aschersonia confluens*. J. Nat. Prod. 84, 1149–1162. doi: 10.1021/acs.jnatprod.0c01212, PMID: 33852304

[ref26] Wang P. Luo Y.-F. Zhang M. Dai J.-G. Wang W.-J. Wu J. (2018). Three xanthone dimers from the Thai mangrove endophytic fungus *Phomopsis* sp. xy21. J. Asian Nat. Prod. Res. 20, 217–226. doi: 10.1016/j.fitote.2018.11.004, PMID: 28581824

[ref27] Wezeman T. Braese S. Masters K.-S. (2015). Xanthone dimers: a compound family which is both common and privileged. Nat. Prod. Rep. 32, 6–28. doi: 10.1039/C4NP00050A, PMID: 25226564

[ref28] Wu J. Wang F. He L.-M. Zhou S.-Y. Wang S.-B. Jia J.. (2022). Aculeaquamide a, cytotoxic paraherquamide from the marine fungus *Aspergillus aculeatinus* WHUF0198. Nat. Prod. Res. 36, 4388–4393. doi: 10.1080/14786419.2021.1998047, PMID: 34720007

[ref29] Wu G. Yu G. Kurtan T. Mandi A. Peng J. Mo X.. (2015). Versixanthones A-F, cytotoxic xanthone-chromanone dimers from the marine-derived fungus *Aspergillus* versicolor HDN1009. J. Nat. Prod. 78, 2691–2698. doi: 10.1021/acs.jnatprod.5b00636, PMID: 26506221

[ref30] Wu J. Zhang H. He L.-M. Xue Y.-Q. Jia J. Wang S.-B.. (2021). A new fusicoccane-type norditerpene and a new indone from the marine-derived fungus *Aspergillus aculeatinus* WHUF0198. Chem. Biodivers. 18:e2100562. doi: 10.1002/cbdv.202100562, PMID: 34382347

[ref31] Xiao Z. Li Y. Gao S. (2017). Total synthesis and structural determination of the dimeric tetrahydroxanthone ascherxanthone A. Org. Lett. 19, 1834–1837. doi: 10.1021/acs.orglett.7b00592, PMID: 28357853

[ref32] Zhang W. Krohn K. Zia-Ullah Florke U. Pescitelli G. Lorenzo D. B.. (2008). New mono-and dimeric members of the secalonic acid family: blennolides A-G isolated from the fungus *Blennoria* sp. Chem. Eur. J. 14, 4913–4923. doi: 10.1002/chem.200800035, PMID: 18425741

[ref33] Zhen X. Gong T. Wen Y.-H. Yan D.-J. Chen J.-J. Zhu P. (2018). Chrysoxanthones A–C, three new Xanthone–Chromanone heterdimers from sponge-associated *Penicillium chrysogenum* HLS111 treated with histone deacetylase inhibitor. Mar. Drugs 16:357. doi: 10.3390/md16100357, PMID: 30275353 PMC6213349

