# Supplementary material for: Aculeaxanthones A–E, new xanthones from the marine-derived fungus Aspergillus aculeatinus WHUF0198
Source: Front Microbiol. 2023 Feb 27;14:1138830. doi: 10.3389/fmicb.2023.1138830 (PMC10008875; doi:10.3389/fmicb.2023.1138830)

*Supplementary Material*

# Aculeaxanthones A−E, New Xanthones from the Marine-Derived Fungus *Aspergillus aculeatinus* WHUF0198

**Jun Wu [1,2,†], Hua Shui [1†], Mengke Zhang [3], Yida Zeng [2], Mingxin Zheng [4], Kong-Kai Zhu [5], Shou-Bao Wang [6], Hongkai Bi [4,*], Kui Hong [2,*], and You-Sheng Cai [1,2,*]**

[1] Department of Nephrology, Zhongnan Hospital of Wuhan University, School of Pharmaceutical Sciences, Wuhan University, Wuhan 430071, China

[2] Key Laboratory of Combinatorial Biosynthesis and Drug Discovery, Ministry of Education and School of Pharmaceutical Sciences, Wuhan University, Wuhan, 430071, China

[3] Department of Pharmacy, Renmin Hospital of Wuhan University, Wuhan 430060, China

[4] Department of Pathogen Biology & Jiangsu Key Laboratory of Pathogen Biology & Helicobacter pylori Research Centre, Nanjing Medical University, Nanjing 211166, China

[5] School of Biological Science and Technology, University of Jinan, Jinan 250022, China

[6] Beijing Key Laboratory of Drug Targets Identification and Drug Screening, Institute of Materia Medica, Chinese Academy of Medical Sciences & Peking Union Medical College, Beijing, 100050, China

*** Correspondence:** kuihong31@whu.edu.cn; hkbi@njmu.edu.cn; cysh2002@whu.edu.cn

[†]These authors contributed equally to this work.

## List of Supporting Information


| No. | | Contents | Page |
|---|---|---|---|
| 1 | **Figure S1** | HRESIMS spectrum of **2.** | S1 |
| 2 | **Figure S2** | $^{1}H$ NMR spectrum of **2** (600 MHz, $CDCl_3$). | S1 |
| 3 | **Figure S3** | $^{13}C$ NMR spectrum of **2** (150 MHz, $CDCl_3$). | S2 |
| 4 | **Figure S4** | Experimental ECD spectrum of **2** (MeOH). | S2 |
| 5 | **Figure S5** | Comparison of the experimental ECD spectrum with the TDDFT-predicted curves of compound **2**. | S3 |
| 6 | **Figure S6** | HRESIMS spectrum of **3.** | S3 |
| 7 | **Figure S7** | $^{1}H$ NMR spectrum of **3** (600MHz, $CD_3OD$). | S4 |
| 8 | **Figure S8** | Experimental ECD spectrum of **3** (MeOH). | S4 |
| 9 | **Figure S9** | Comparison of the experimental ECD spectrum with the TDDFT-predicted curves of compound **3**. | S5 |
| 10 | **Figure S10** | HRESIMS spectrum of **4.** | S5 |
| 11 | **Figure S11** | $^{1}H$ NMR spectrum of **4** (600 MHz, $CDCl_3$). | S6 |
| 12 | **Figure S12** | $^{1}H$-$^{1}H$ COSY spectrum of **4** (600 MHz, $CDCl_3$). | S6 |
| 13 | **Figure S13** | HSQC spectrum of **4** (600 MHz, $CDCl_3$). | S7 |
| 14 | **Figure S14** | HMBC spectrum of **4** (600 MHz, $CDCl_3$). | S7 |
| 15 | **Figure S15** | NOESY spectrum of **4** (600 MHz, $CDCl_3$). | S8 |
| 16 | **Figure S16** | $^{1}H$ NMR spectrum of conversion of **2** to **4** (400 MHz, DMSO-$d_6$). | S8 |
| 17 | **Figure S17** | HPLC analysis of conversion of **2** to **4** in DMSO-$d_6$. | S9 |
| 18 | **Figure S18** | $^{1}H$ NMR spectrum of conversion and isolation of **4** (600 MHz, $CDCl_3$). | S9 |
| 19 | **Figure S19** | Experimental ECD spectrum of **4** (MeOH). | S10 |
| 20 | **Figure S20** | HRESIMS spectrum of **5**. | S10 |
| 21 | **Figure S21** | $^{1}H$ NMR spectrum of **5** (600 MHz, $CDCl_3$). | S11 |
| 22 | **Figure S22** | $^{1}H$-$^{1}H$ COSY spectrum of **5** (600 MHz, $CDCl_3$). | S11 |
| 23 | **Figure S23** | HSQC spectrum of **5** (600 MHz, $CDCl_3$). | S12 |
| 24 | **Figure S24** | HMBC spectrum of **5** (600 MHz, $CDCl_3$). | S12 |
| 25 | **Figure S25** | NOESY spectrum of **5** (600 MHz, $CDCl_3$). | S13 |
| 26 | **Figure S26** | Experimental ECD spectrum of **5** (MeOH). | S13 |
| 27 | **Figure S27** | HRESIMS spectrum of **6**. | S14 |
| 28 | **Figure S28** | $^{1}H$ NMR spectrum of **6** (600 MHz, $CDCl_3$). | S14 |
| 29 | **Figure S29** | $^{1}H$-$^{1}H$ COSY spectrum of **6** (600 MHz, $CDCl_3$). | S15 |
| 30 | **Figure S30** | HSQC spectrum of **6** (600 MHz, $CDCl_3$). | S15 |
| 31 | **Figure S31** | HMBC spectrum of **6** (600 MHz, $CDCl_3$). | S16 |

| | | | |
|---|---|---|---|
| 32 | **Figure S32** | NOESY spectrum of **6** (600 MHz, $CDCl_3$). | S16 |
| 33 | **Figure S33** | Experimental ECD spectrum of **6** (MeOH). | S17 |
| 34 | **Figure S34** | HRESIMS spectrum of **7**. | S17 |
| 35 | **Figure S35** | $^1$H NMR spectrum of **7** (600 MHz, $CDCl_3$). | S18 |
| 36 | **Figure S36** | $^1$H-$^1$H COSY spectrum of **7** (600 MHz, $CDCl_3$). | S18 |
| 37 | **Figure S37** | HSQC spectrum of **7** (600 MHz, $CDCl_3$). | S19 |
| 38 | **Figure S38** | HMBC spectrum of **7** (600 MHz, $CDCl_3$). | S19 |
| 39 | **Figure S39** | NOESY spectrum of **7** (600 MHz, $CDCl_3$). | S20 |
| 40 | **Figure S40** | Experimental ECD spectrum of **7** (MeOH). | S20 |
| 41 | **Figure S41** | HRESIMS spectrum of **8**. | S21 |
| 42 | **Figure S42** | $^1$H NMR spectrum of **8** (600 MHz, $CDCl_3$). | S21 |
| 43 | **Figure S43** | $^{13}$C NMR spectrum of **8** (150 MHz, $CDCl_3$). | S22 |
| 44 | **Figure S44** | $^1$H-$^1$H COSY spectrum of **8** (600 MHz, $CDCl_3$). | S22 |
| 45 | **Figure S45** | HSQC spectrum of **8** (600 MHz, $CDCl_3$). | S23 |
| 46 | **Figure S46** | HMBC spectrum of **8** (600 MHz, $CDCl_3$). | S23 |
| 47 | **Figure S47** | NOESY spectrum of **8** (600 MHz, $CDCl_3$). | S24 |
| 48 | **Figure S48** | Experimental ECD spectrum of **8** (MeOH). | S24 |
| 49 | **Figure S49** | HRESIMS spectrum of **9**. | S25 |
| 50 | **Figure S50** | $^1$H NMR spectrum of **9** (600 MHz, $CDCl_3$). | S25 |
| 51 | **Figure S51** | Experimental ECD spectrum of **9** (MeOH). | S26 |
| 52 | **Figure S52** | Comparison of the experimental ECD spectrum with the TDDFT-predicted curves of compound **3**. | S26 |

**Figure S1** HRESIMS spectrum of **2.**

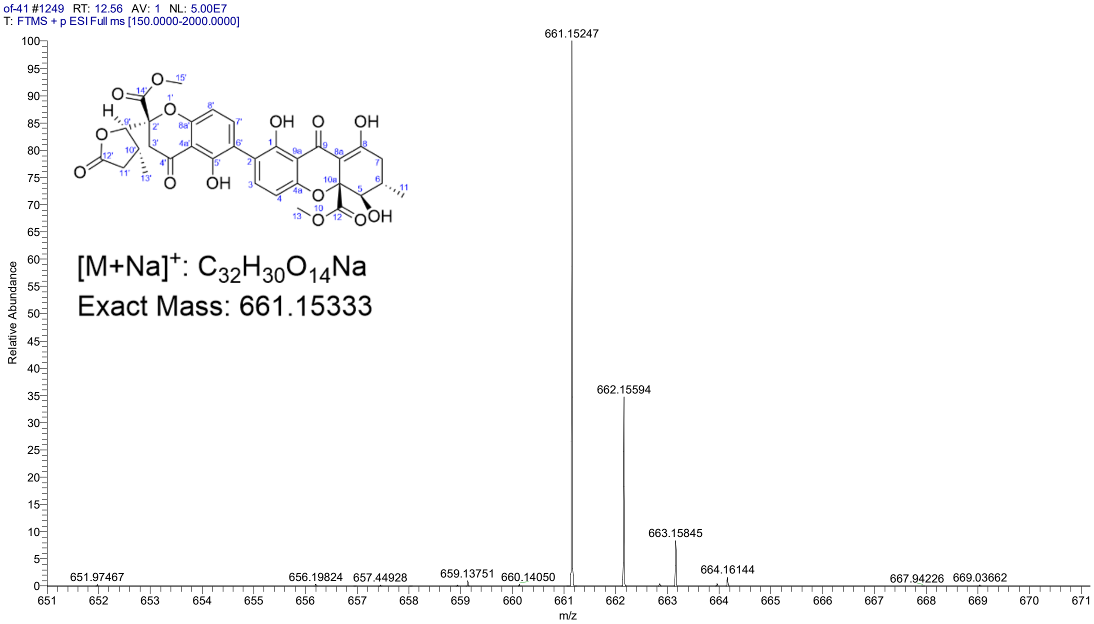


**Figure S2** $^{1}$H NMR spectrum of **2** (600 MHz, $CDCl_3$).

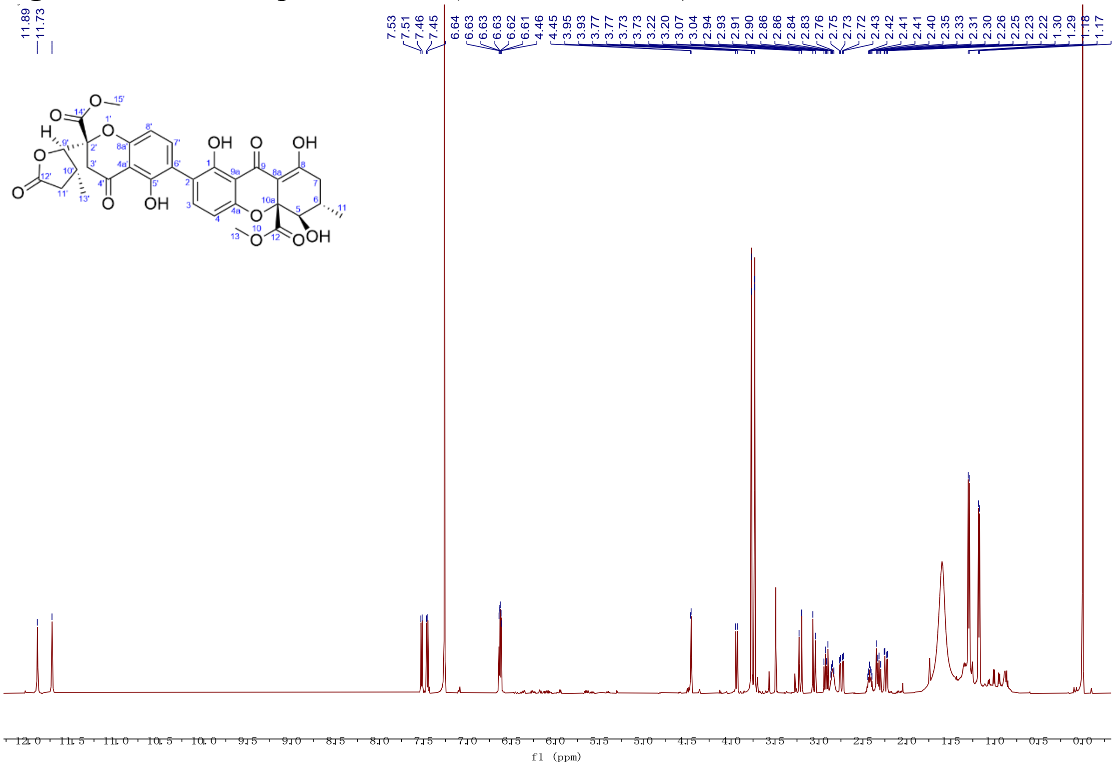

**Figure S3** $^{13}$C NMR spectrum of **2** (150 MHz, $CDCl_3$).

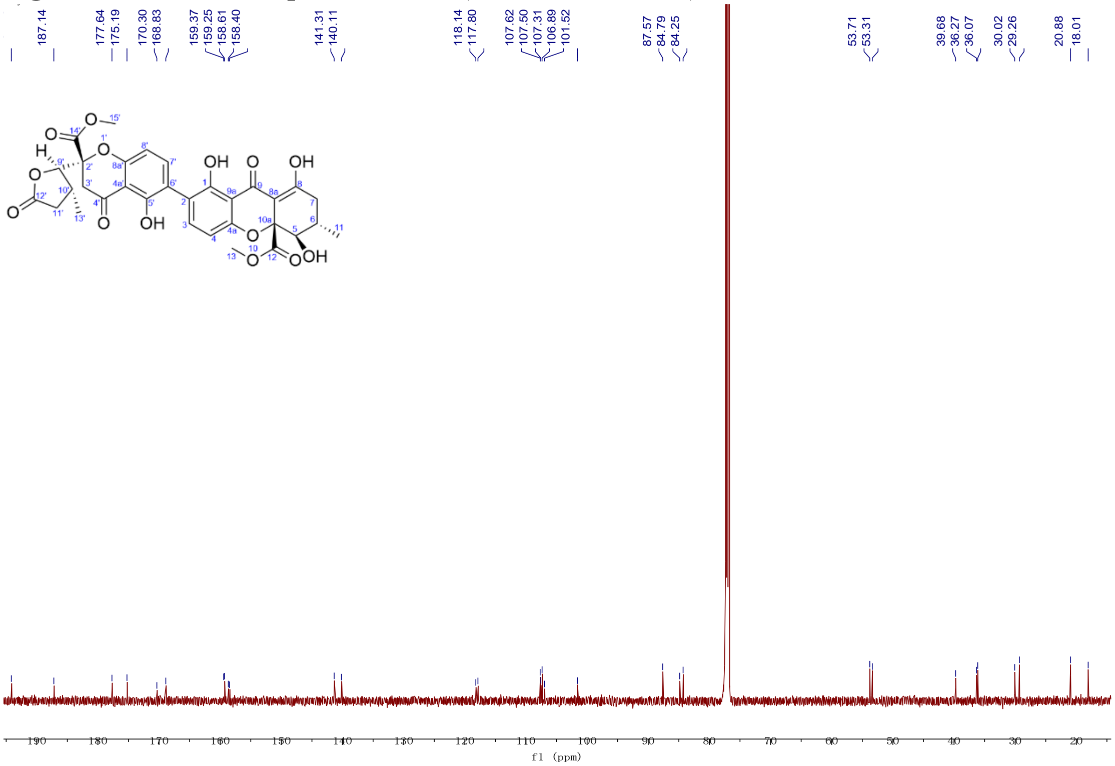


**Figure S4** Experimental ECD spectrum of **2** (MeOH).

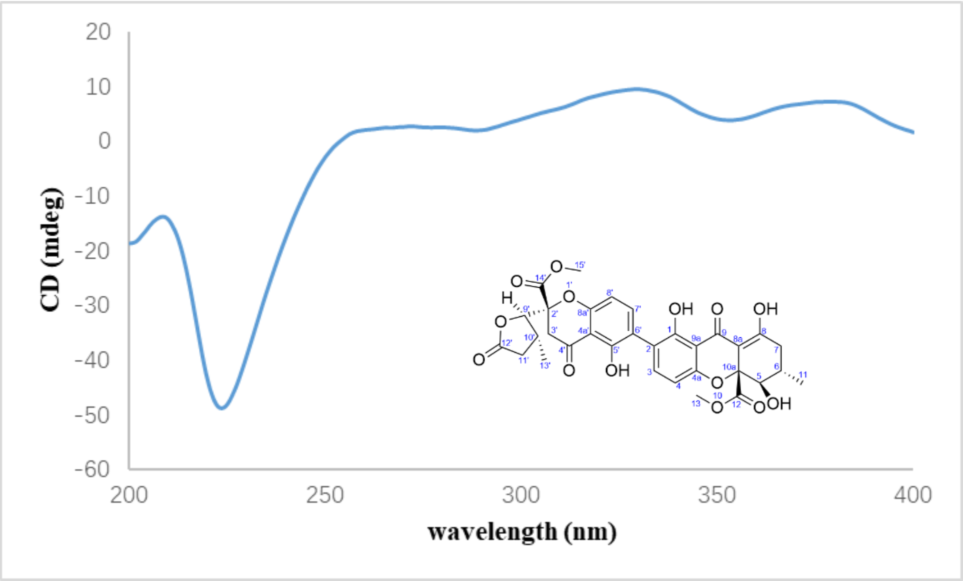

**Figure S5.** Comparison of the experimental ECD spectrum with the TDDFT-predicted curves of compound **2**.

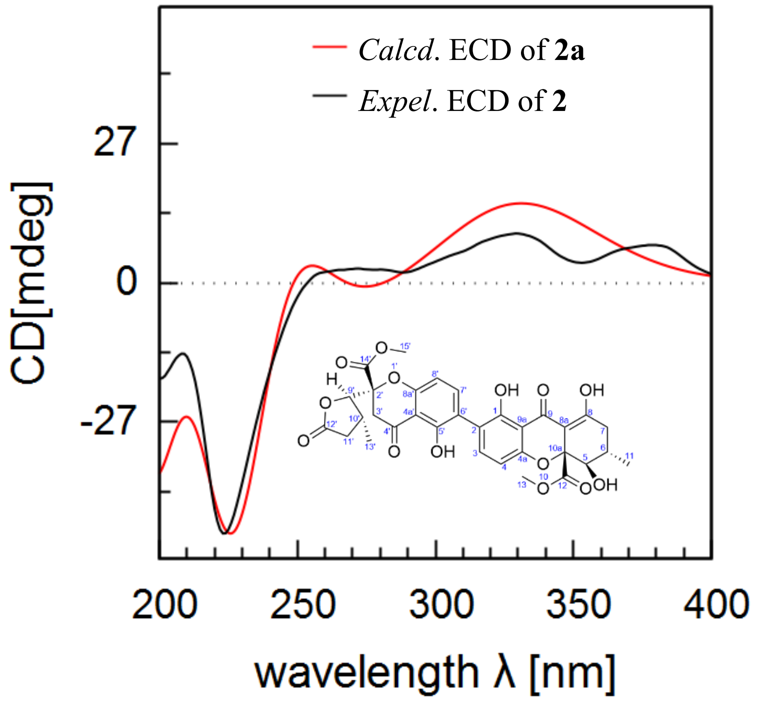


**Figure S6** HRESIMS spectrum of **3**.

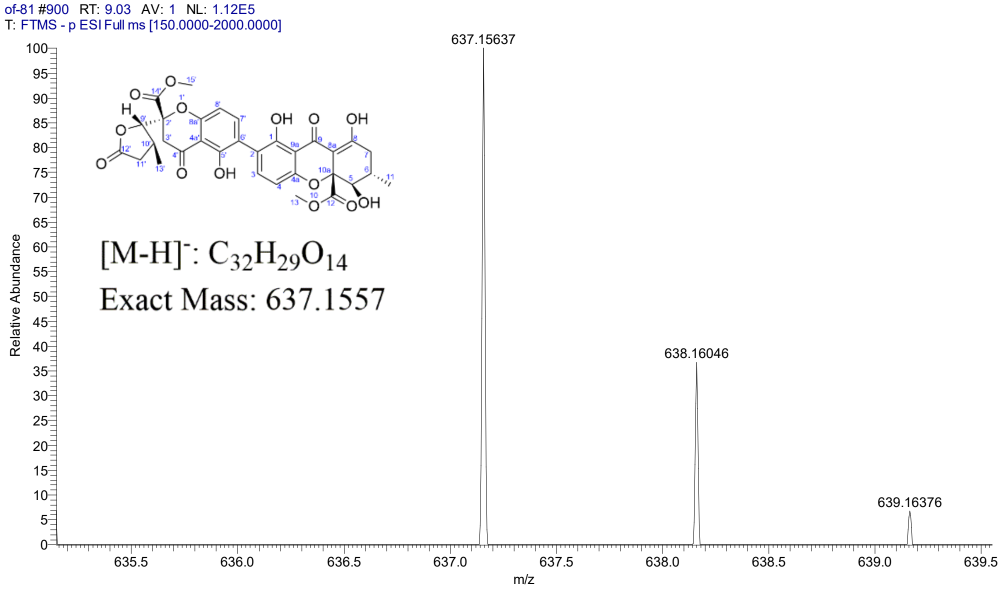

**Figure S7** $^{1}$H NMR spectrum of **3** (600 MHz, $CDCl_3$).

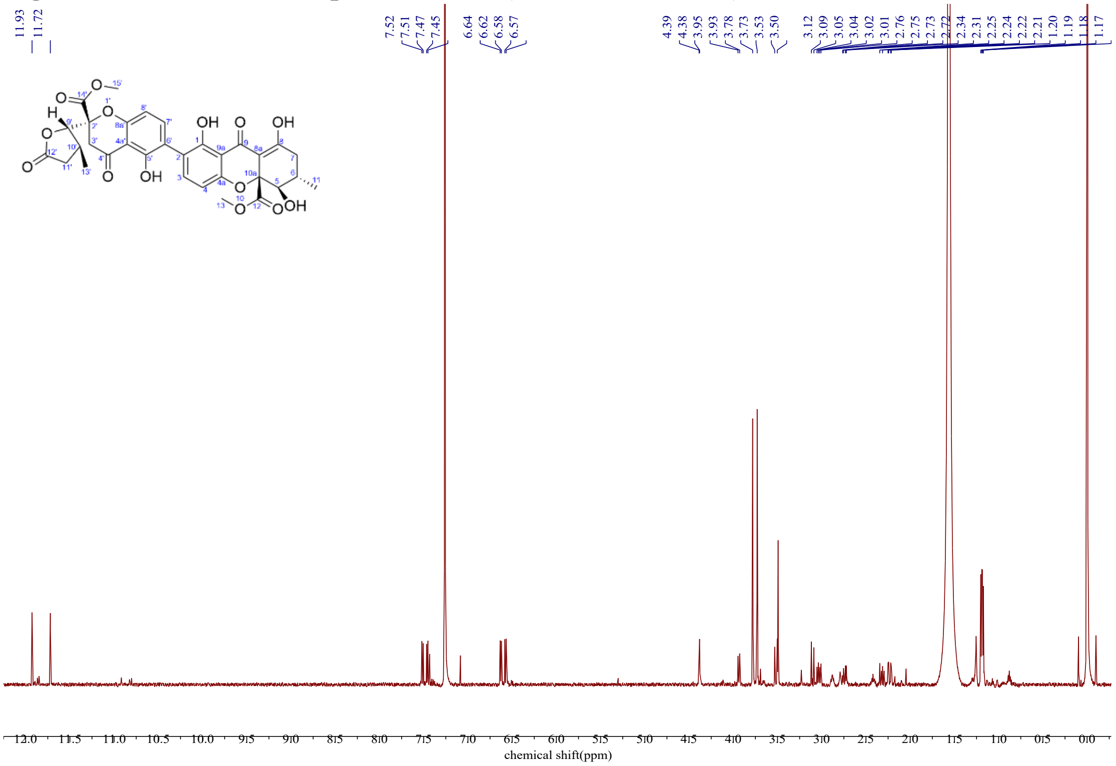

**Figure S8** Experimental ECD spectrum of **3** (MeOH).

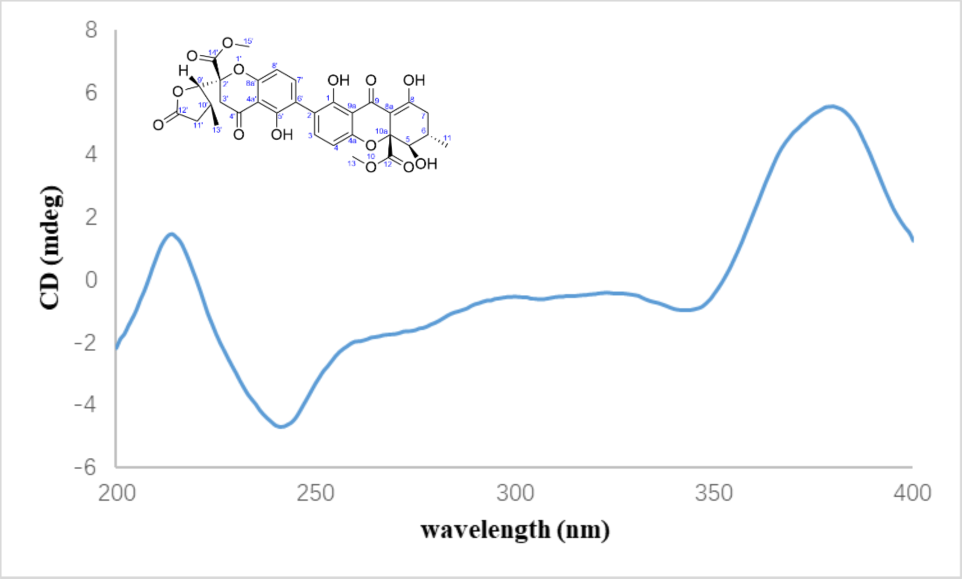

**Figure S9** Comparison of the experimental ECD spectrum with the TDDFT-predicted curves of compound **3**.

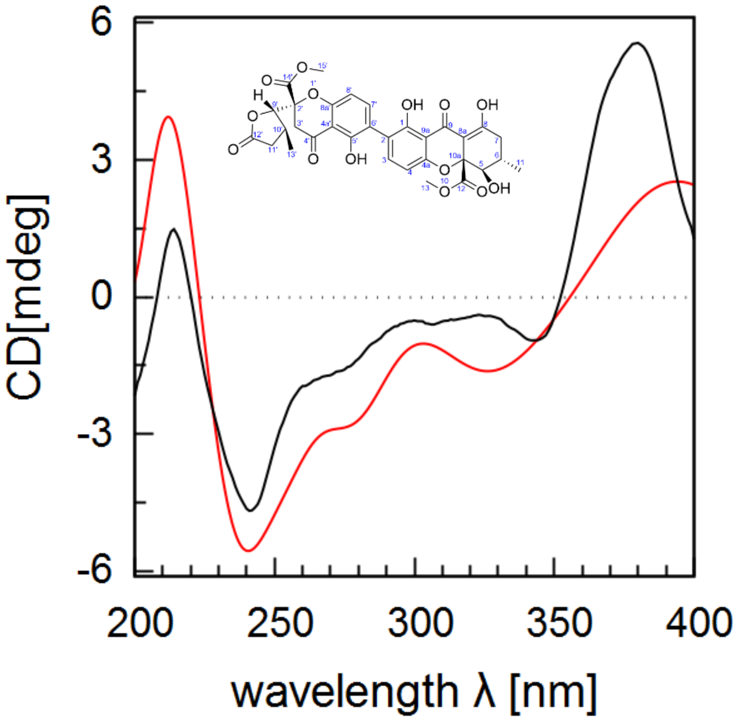


**Figure S10** HRESIMS spectrum of **4**.

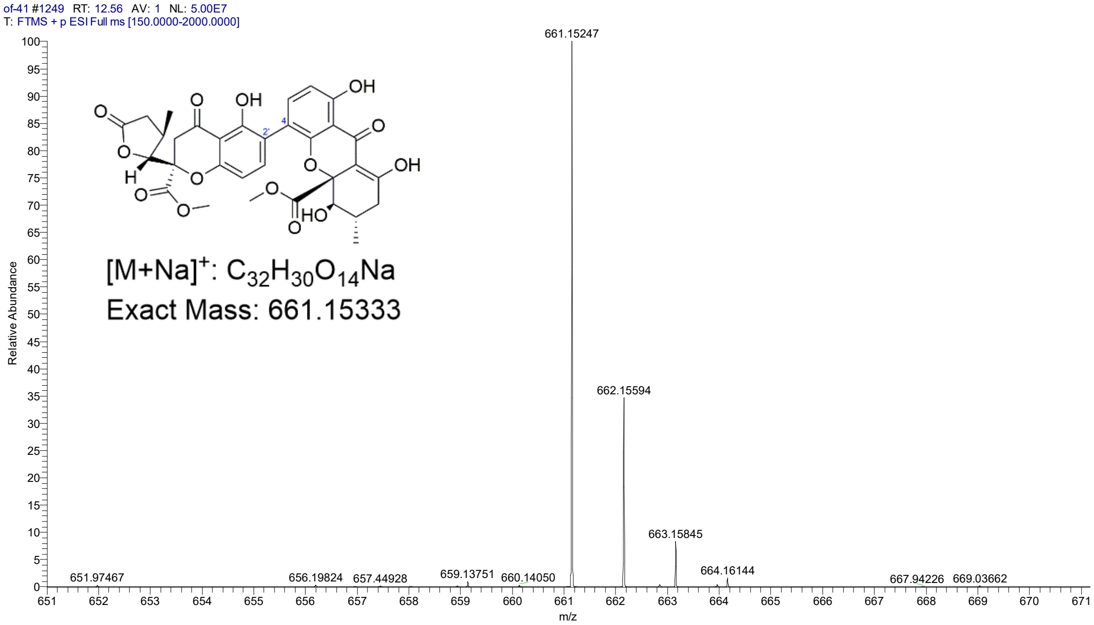

**Figure S11** $^1$H NMR spectrum of **4** (600 MHz, $CDCl_3$).

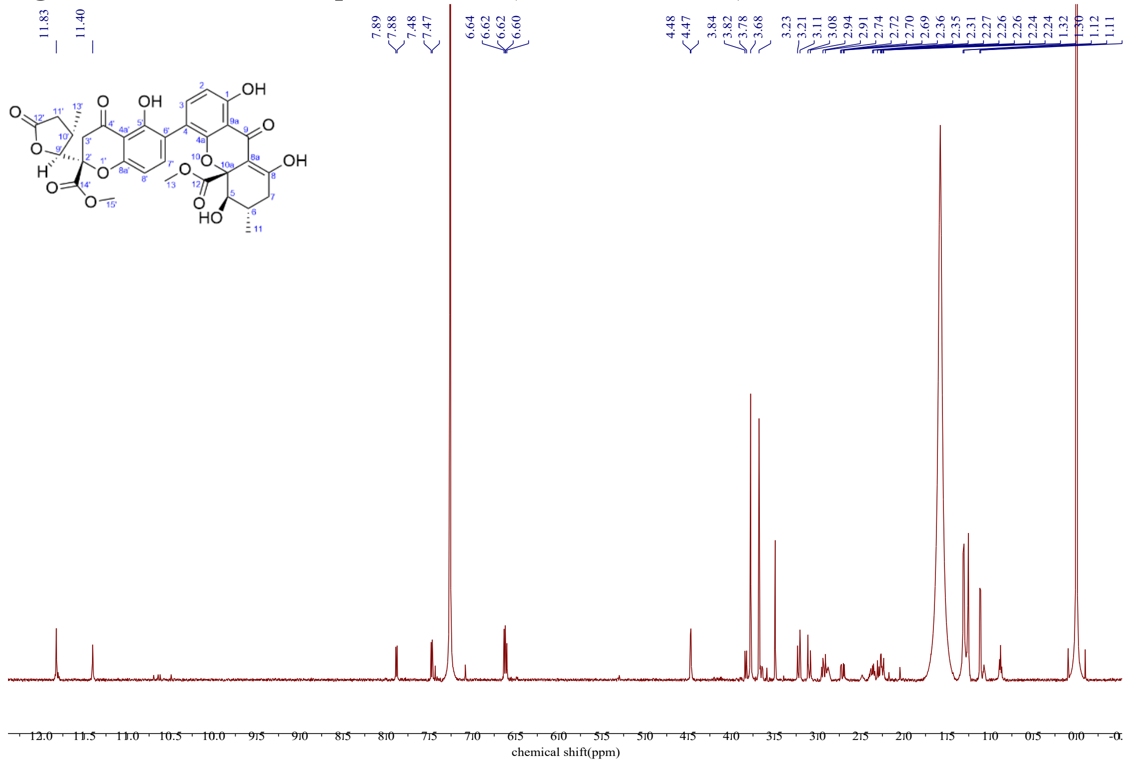

**Figure S12** $^1$H-$^1$H COSY spectrum of **4** (600 MHz, $CDCl_3$).

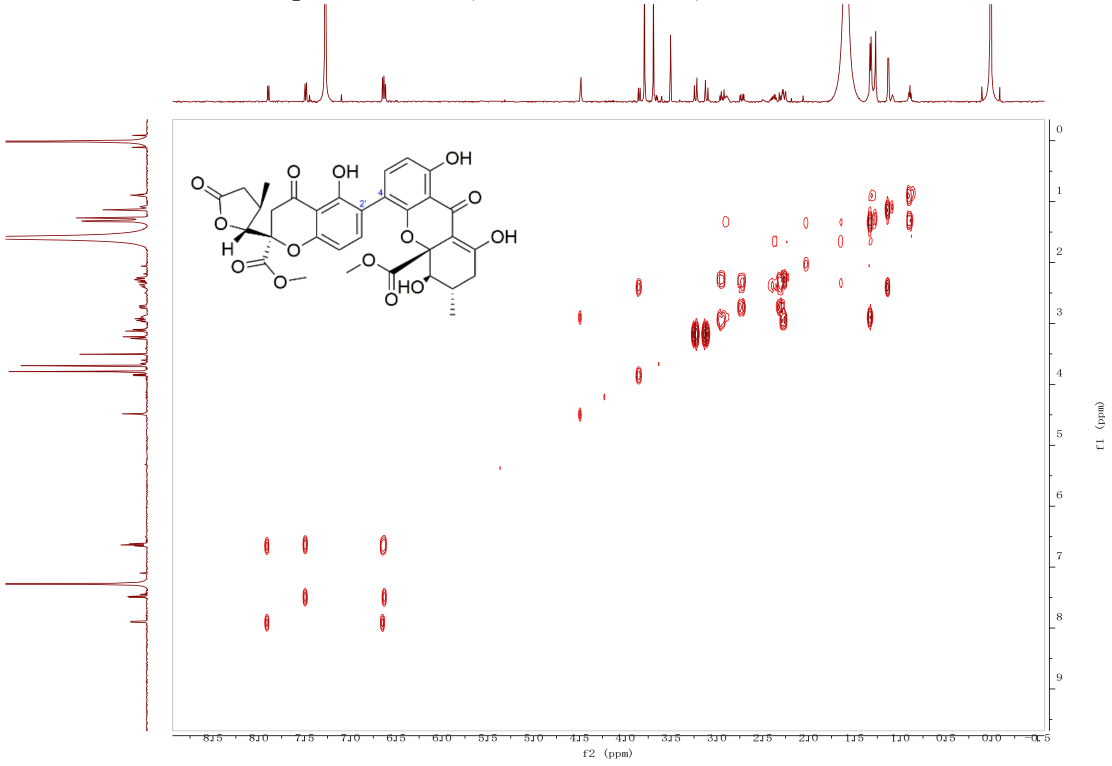

**Figure S13** HSQC spectrum of **4** (600 MHz, $CDCl_3$).

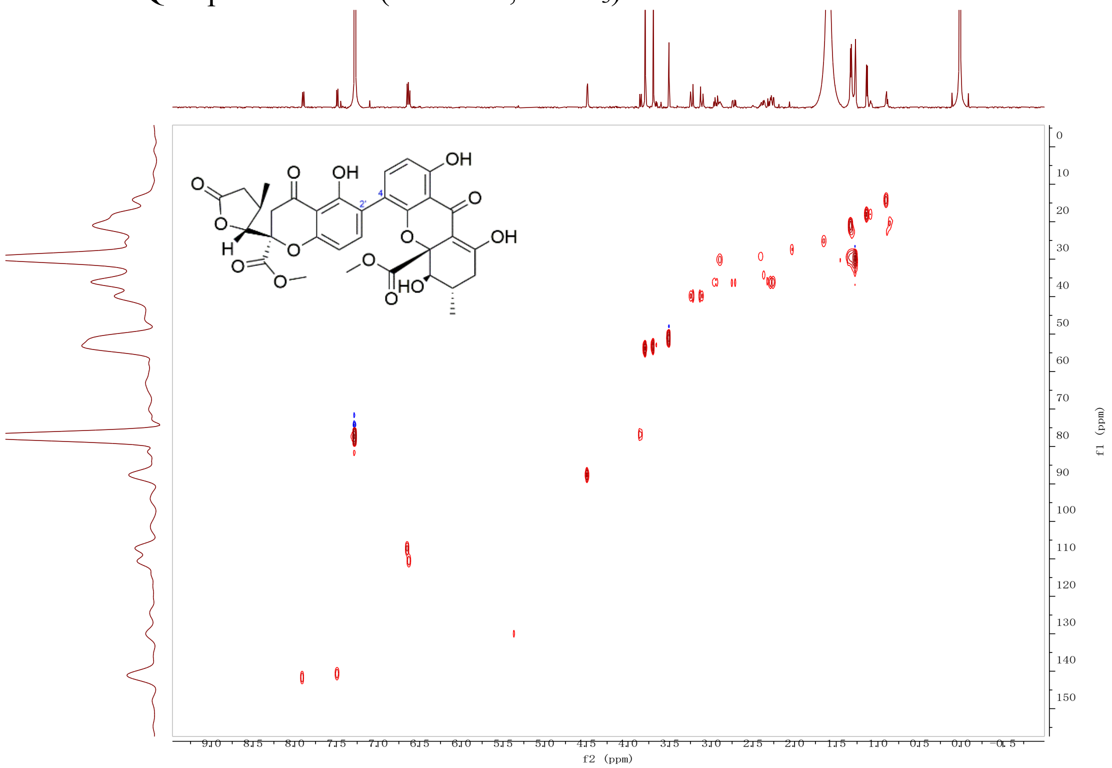


**Figure S14** HMBC spectrum of **4** (600 MHz, $CDCl_3$).

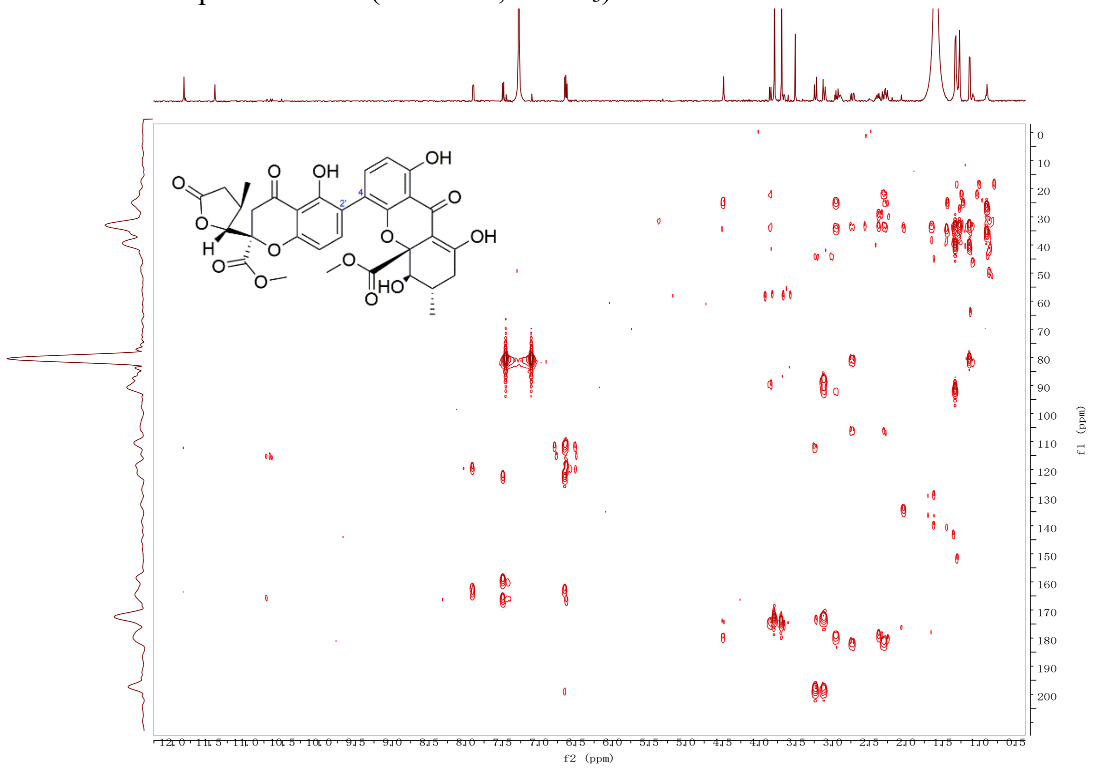

**Figure S15** NOESY spectrum of **4** (600 MHz, $CDCl_3$).

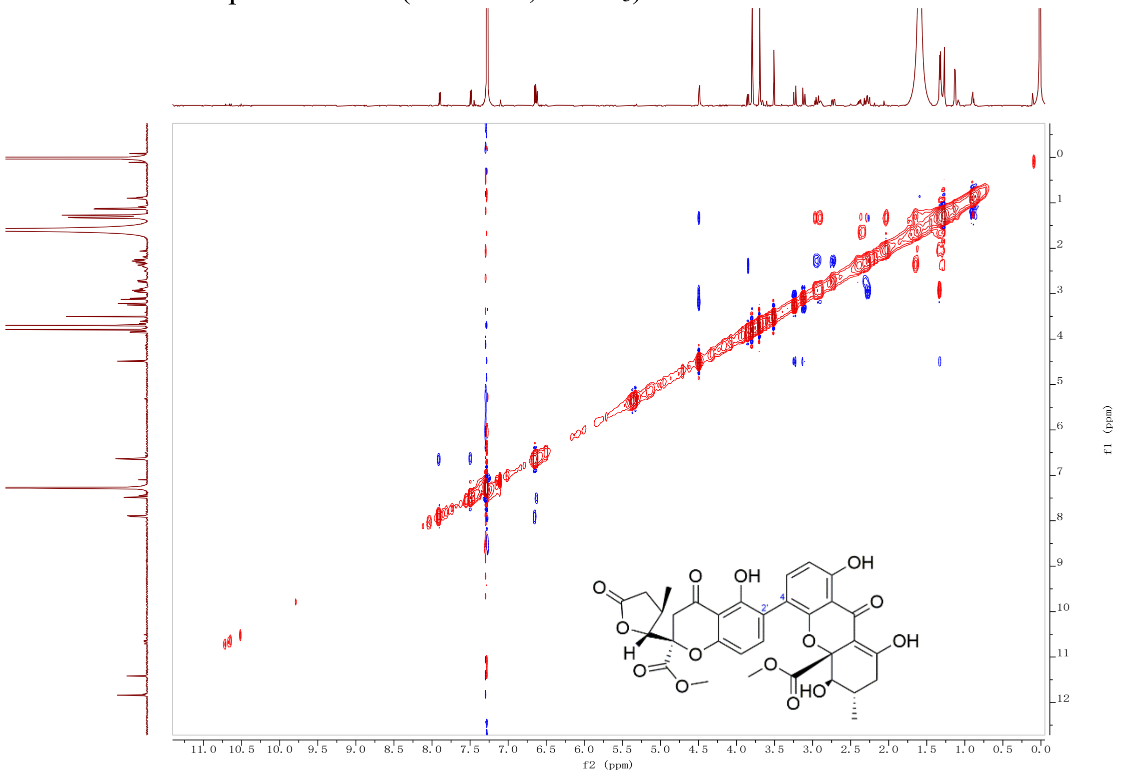

**Figure S16** $^1$H NMR spectrum of conversion of **2** to **4** (400 MHz, DMSO-$d_6$).

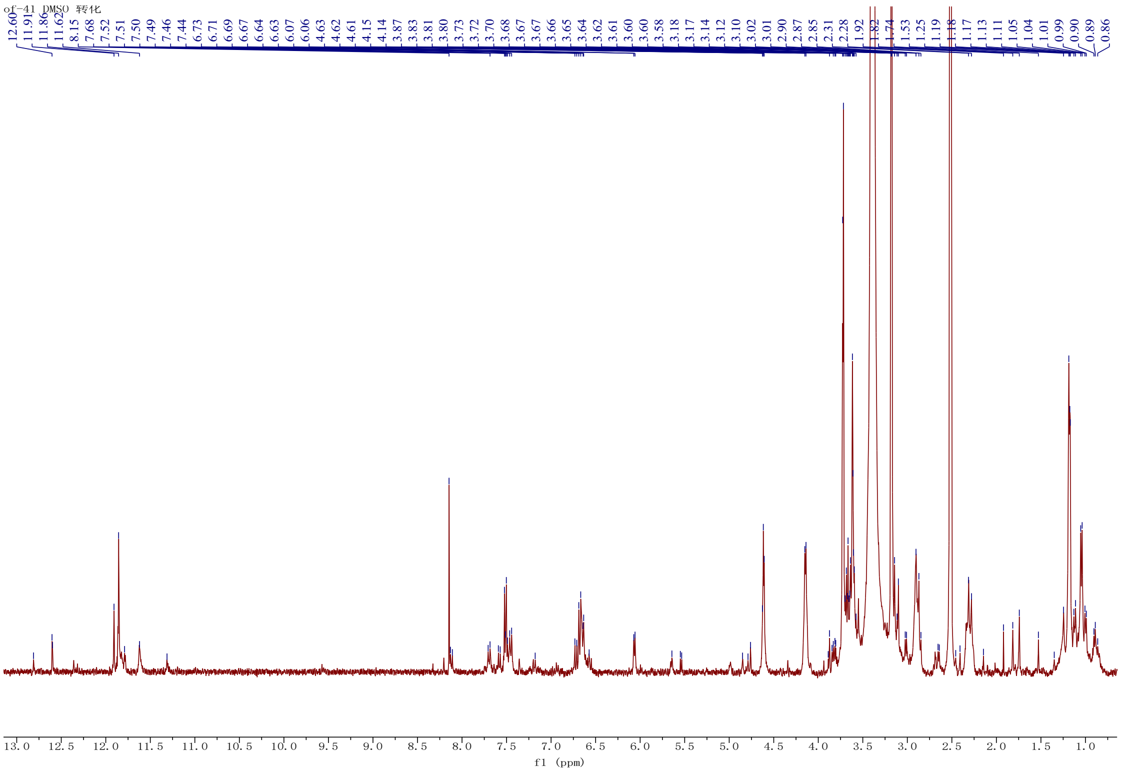

**Figure S17** HPLC analysis of conversion of **2** to **4** (**3**) in DMSO-$d_6$.

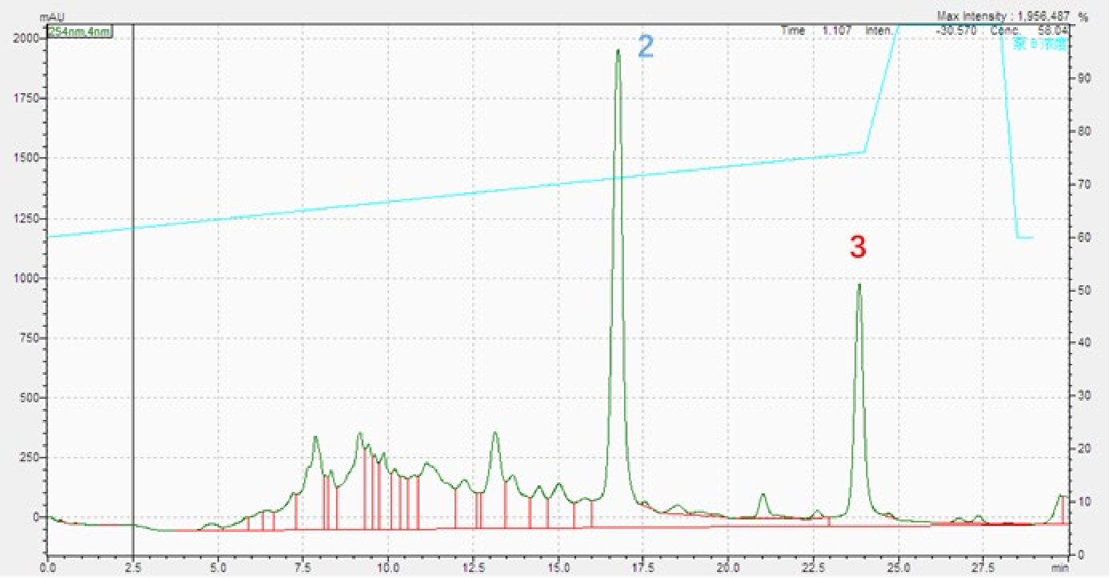


**Figure S18** $^{1}$H NMR spectrum of conversion and isolation of **4** (600 MHz, $CDCl_3$).

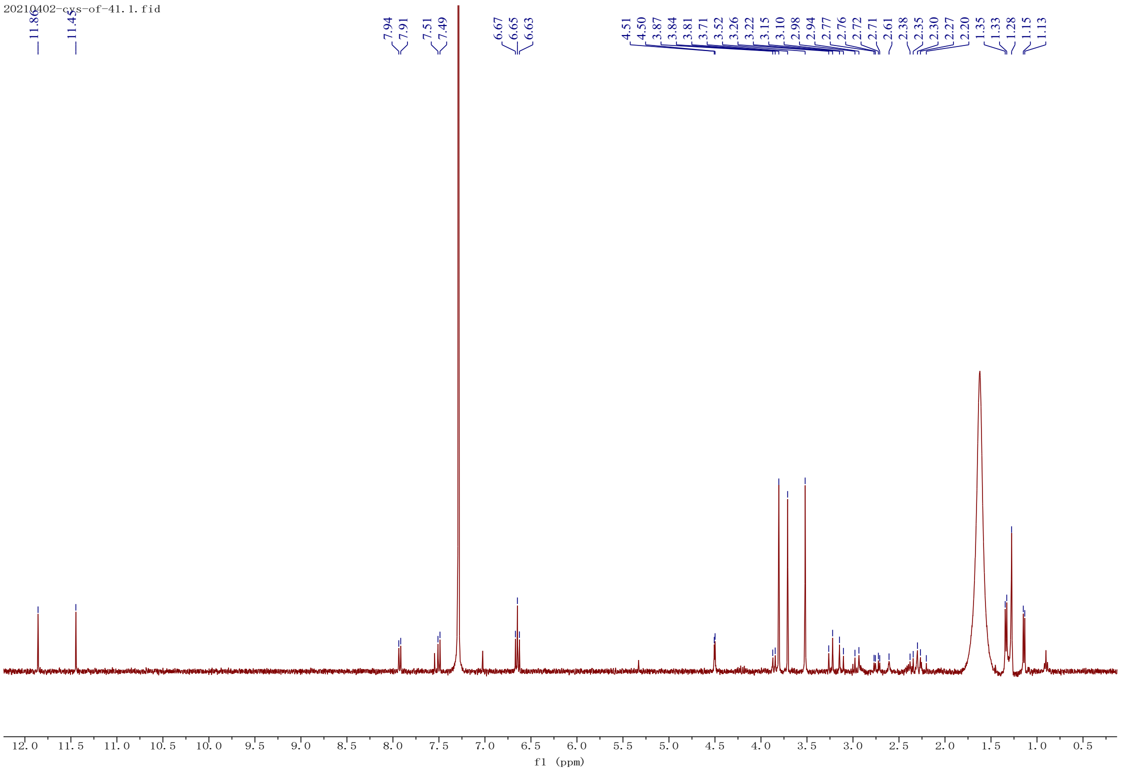

**Figure S19** Experimental ECD spectrum of **4** (MeOH).

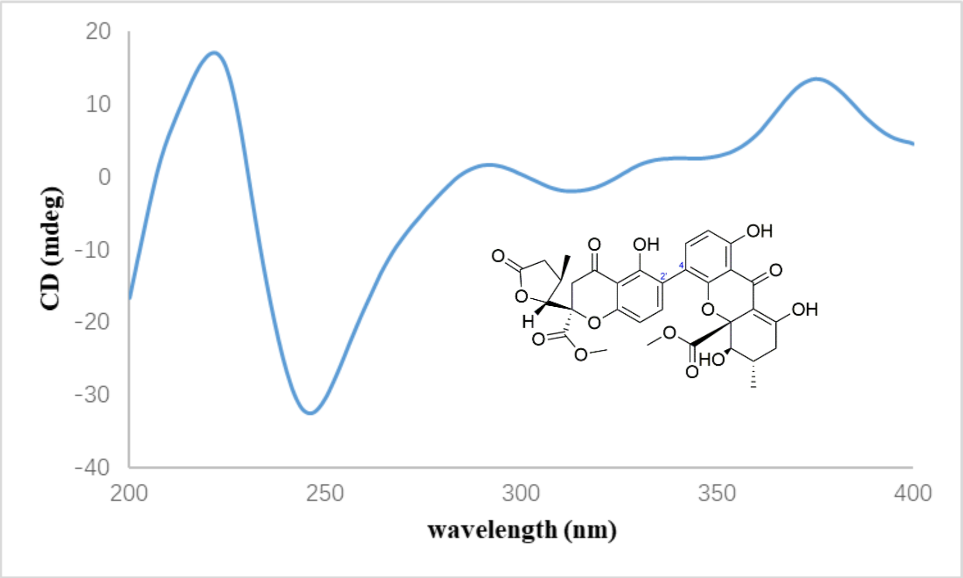


**Figure S20** HRESIMS spectrum of **5.**

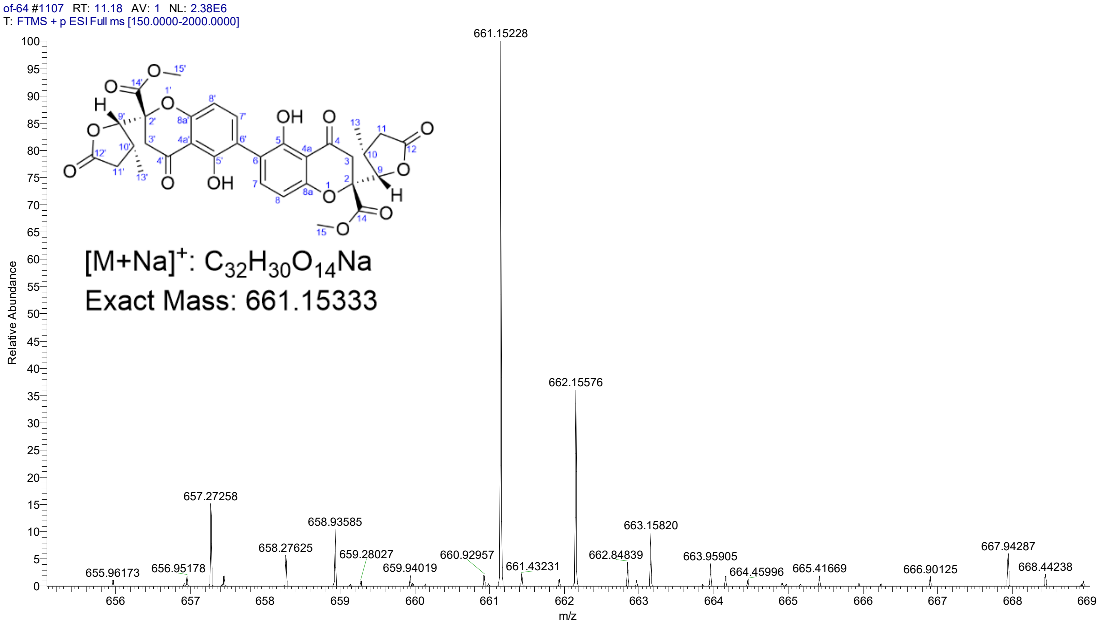

**Figure S21** $^{1}$H NMR spectrum of **5** (600 MHz, $CDCl_3$).

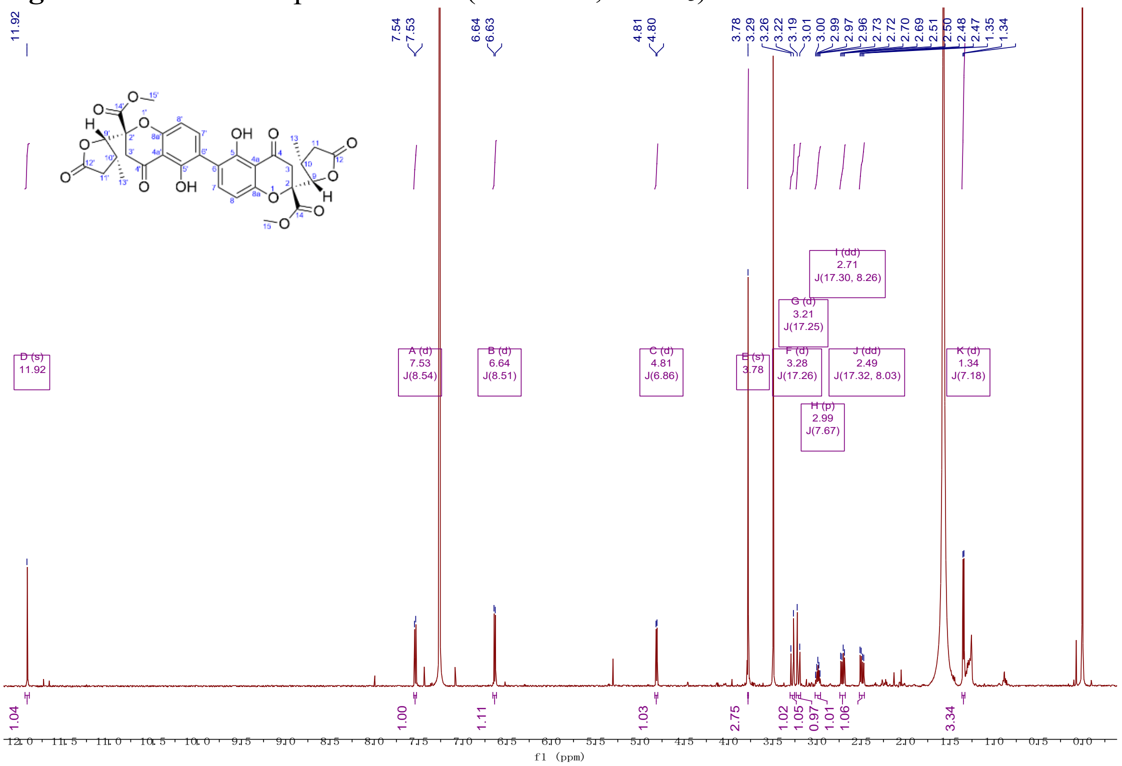

**Figure S22** HSQC spectrum of **5** (600 MHz, $CDCl_3$).

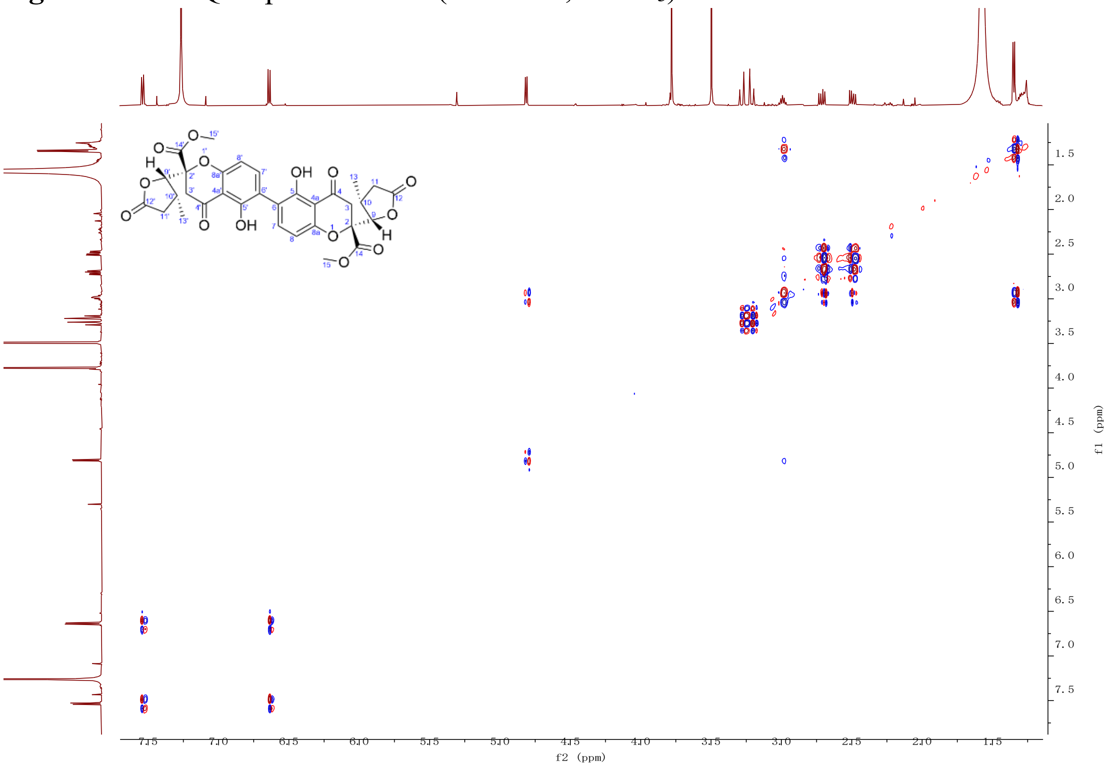

**Figure S23** HSQC spectrum of **5** (600 MHz, $CDCl_3$).

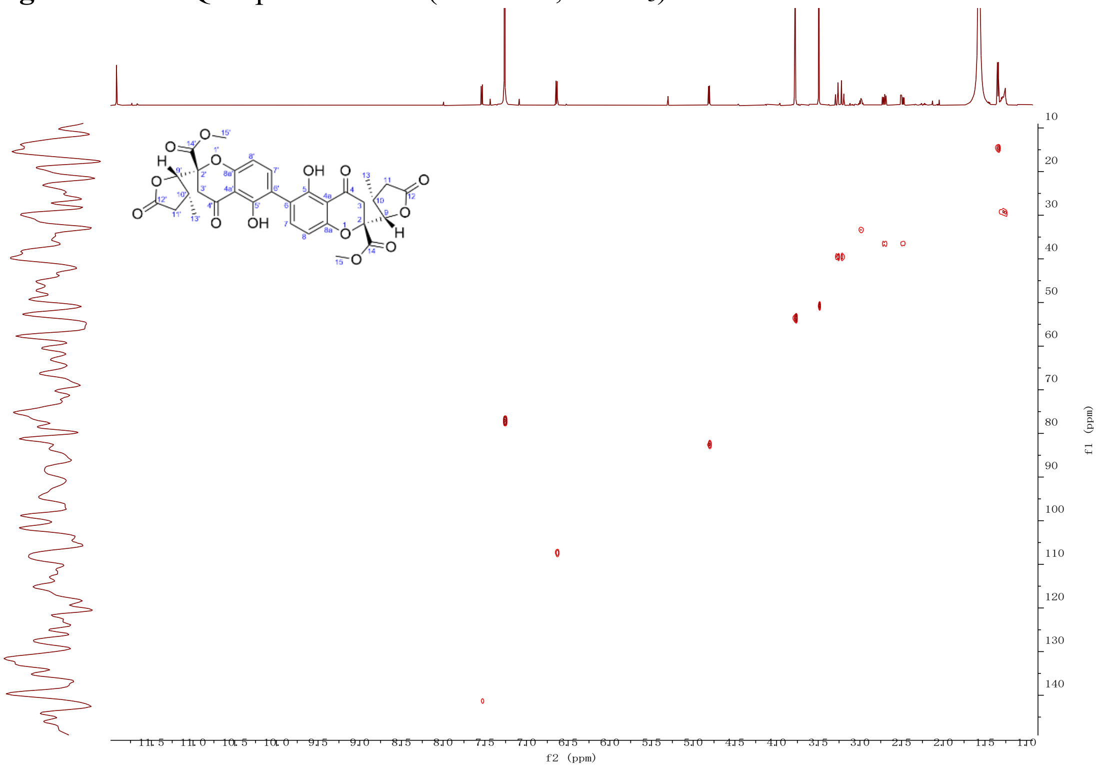

**Figure S24** HMBC spectrum of **5** (600 MHz, $CDCl_3$).

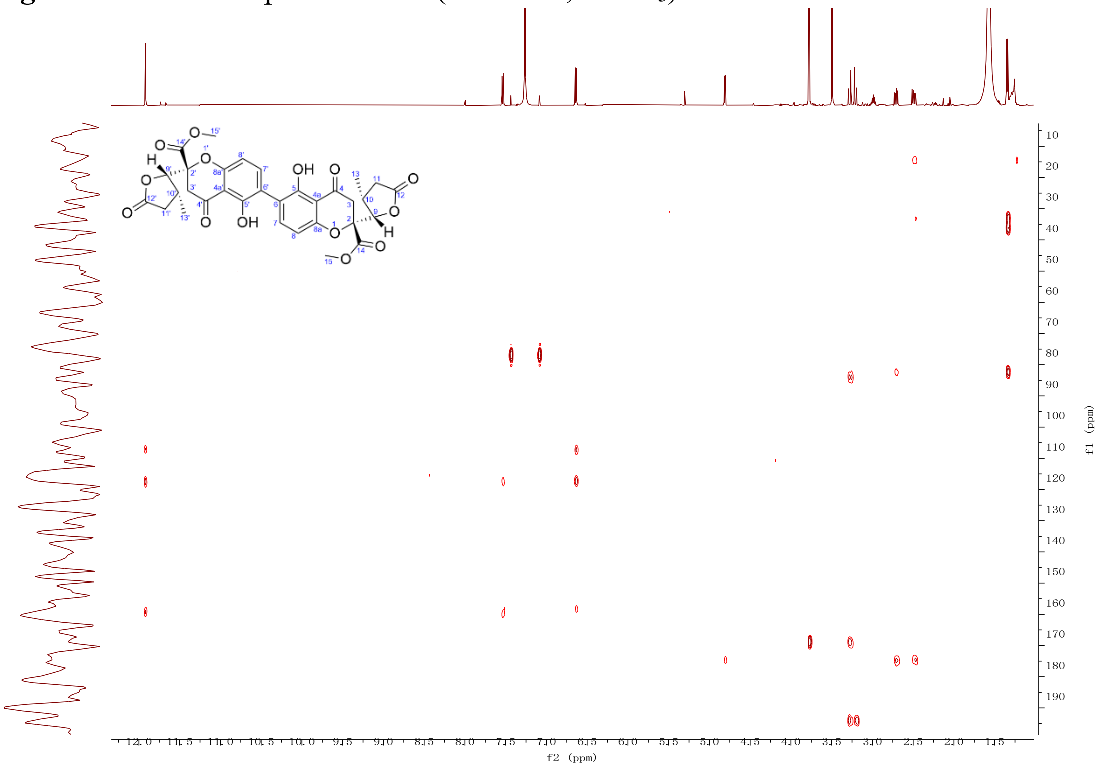

**Figure S25** NOESY spectrum of **5** (600 MHz, $CDCl_3$).

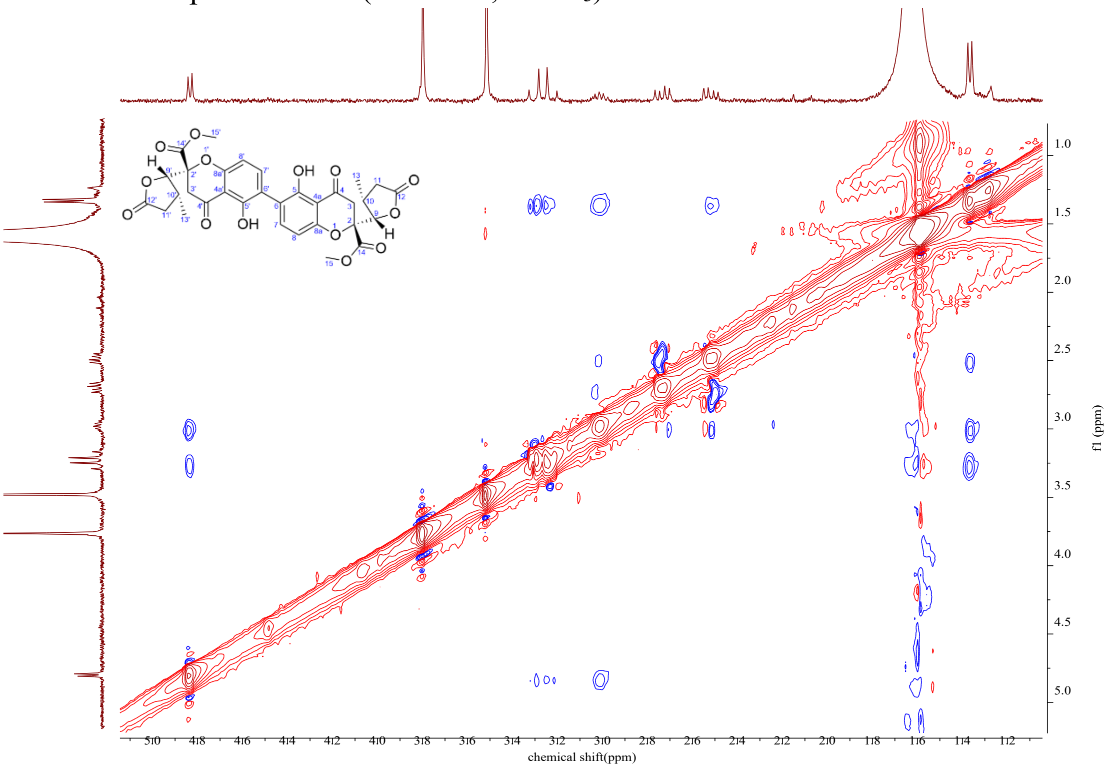


**Figure S26** experimental ECD spectrum of **5** (MeOH).

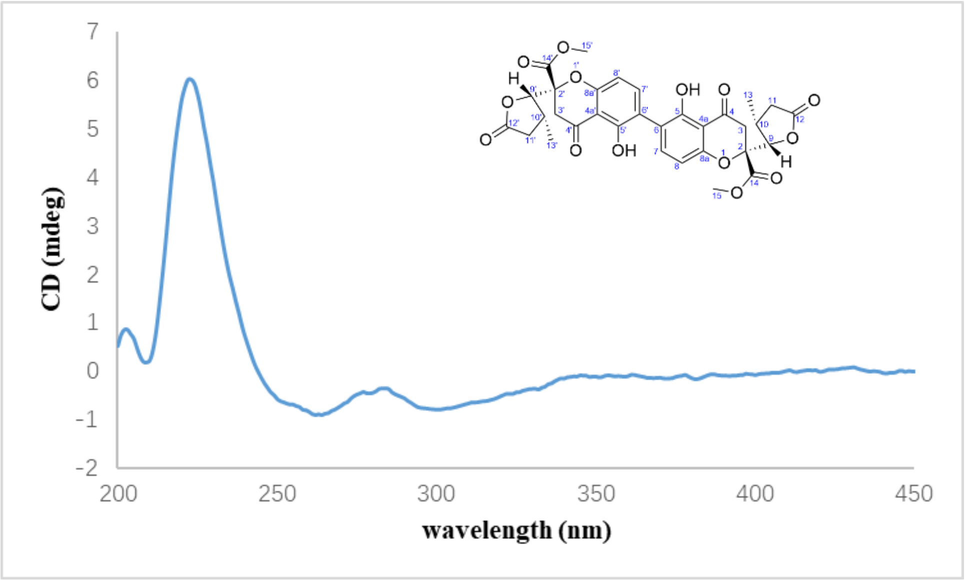

**Figure S27** HRESIMS spectrum of **6**.

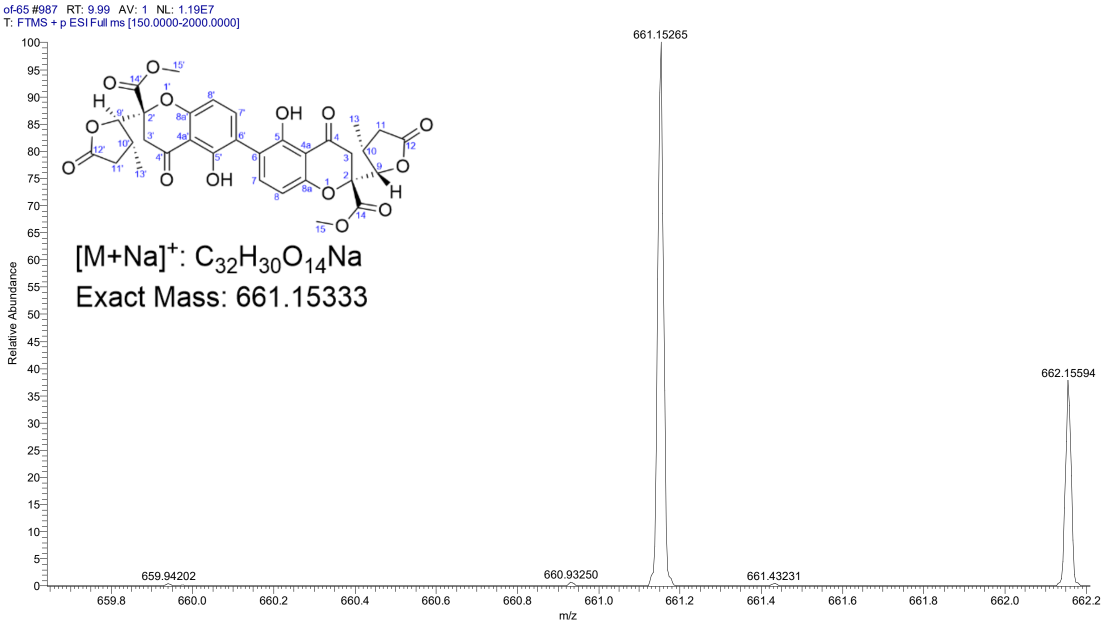


**Figure S28** $^{1}$H NMR spectrum of **6** (600 MHz, $CDCl_3$).

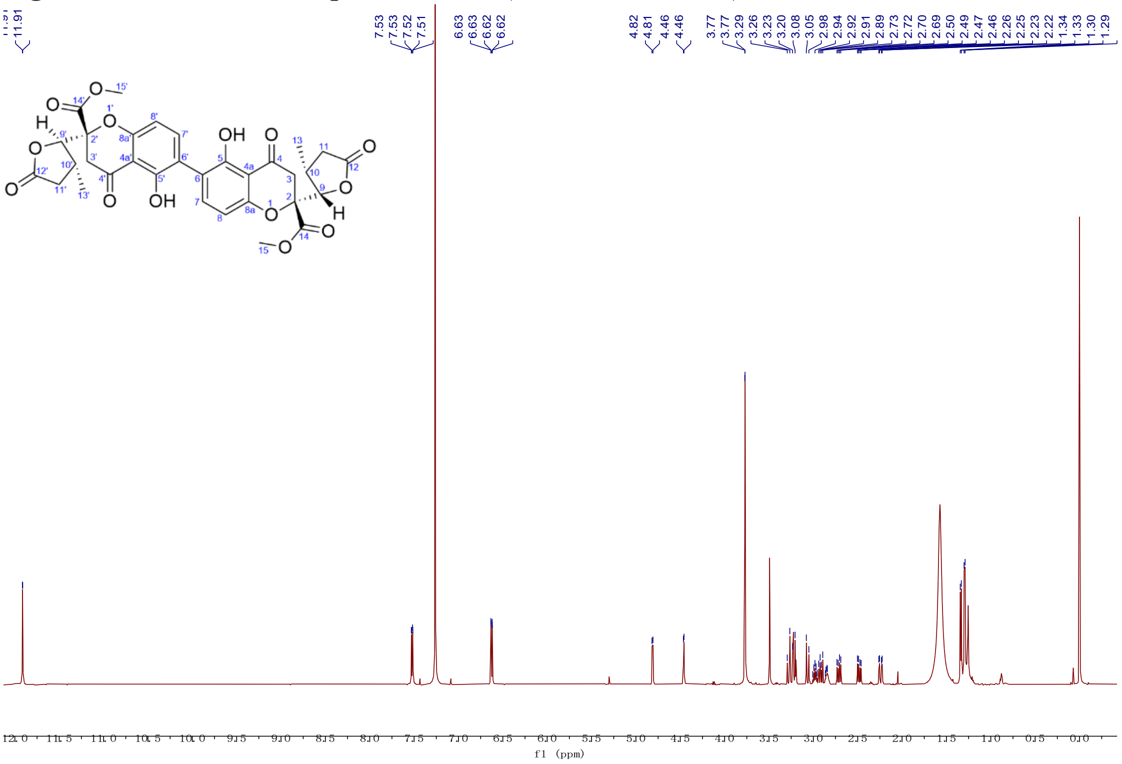

**Figure S29** $^1H$-$^1H$ COSY spectrum of **6** (600 MHz, $CDCl_3$).

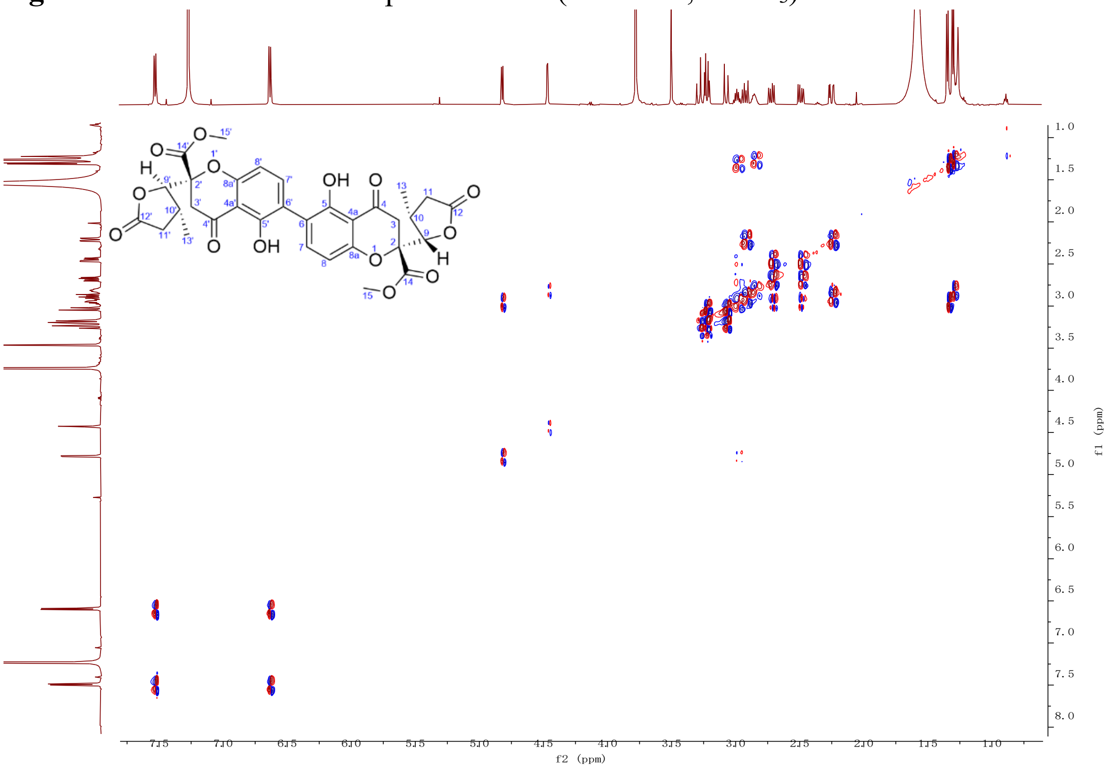

**Figure S30** HSQC spectrum of **6** (600 MHz, $CDCl_3$).

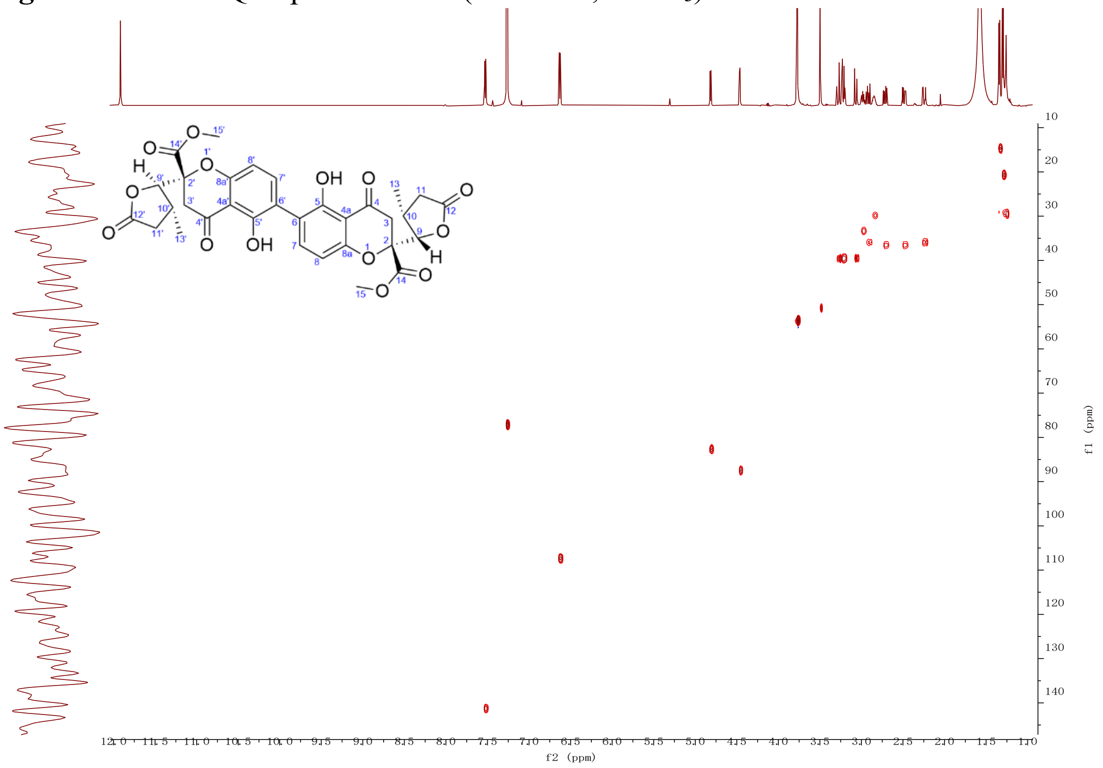

**Figure S31** HMBC spectrum of **6** (600 MHz, $CDCl_3$).

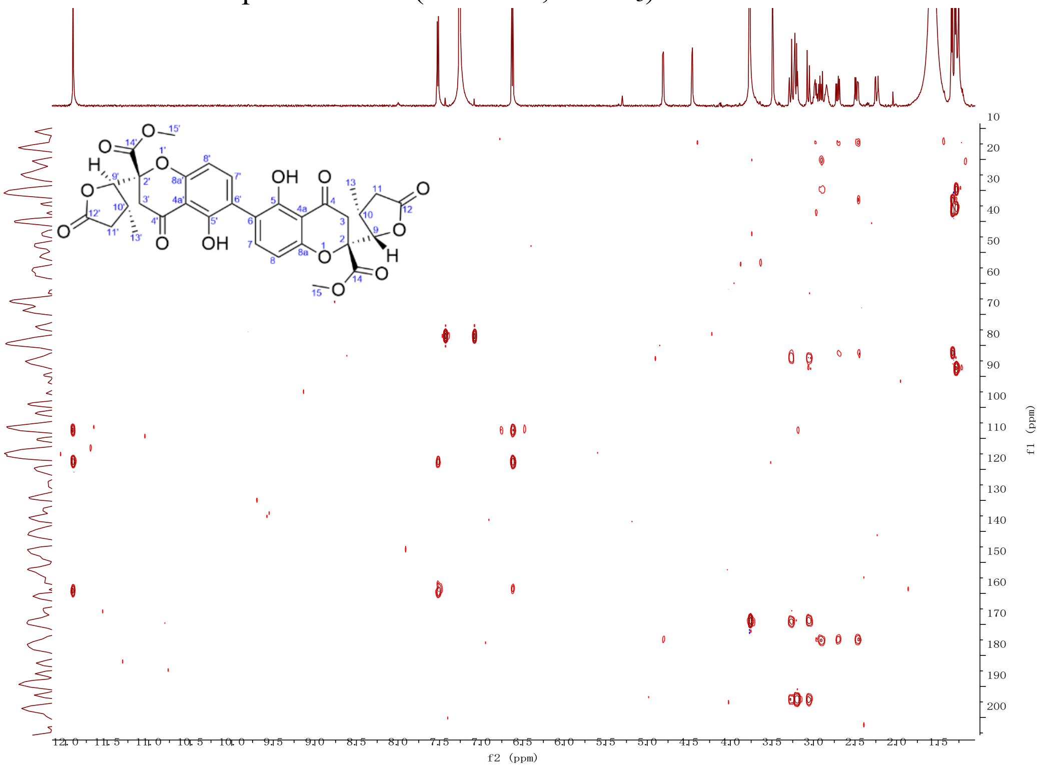

**Figure S32** NOESY spectrum of **6** (600 MHz, $CDCl_3$).

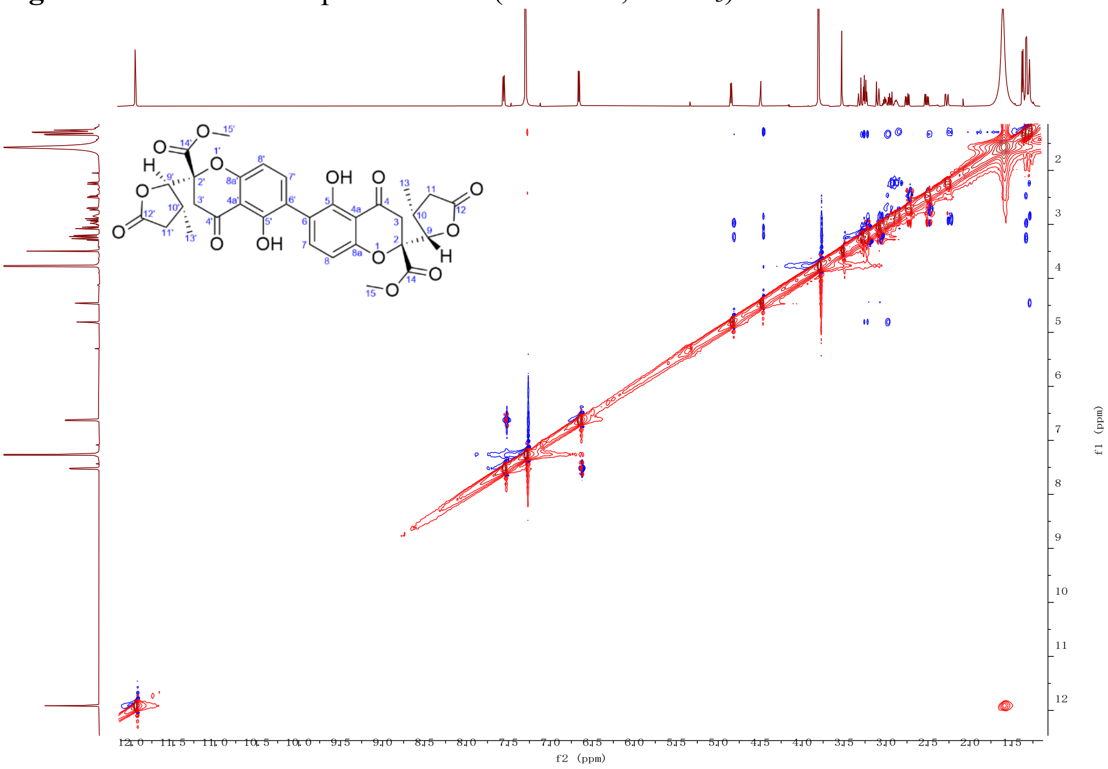

**Figure S33** Experimental ECD spectrum of **6** (MeOH).

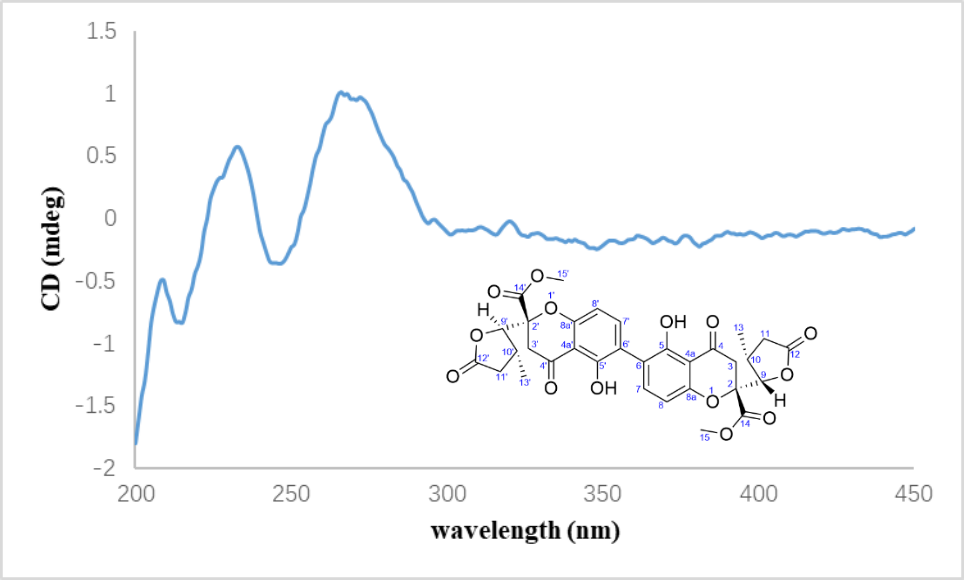


**Figure S34** HRESIMS spectrum of **7**.

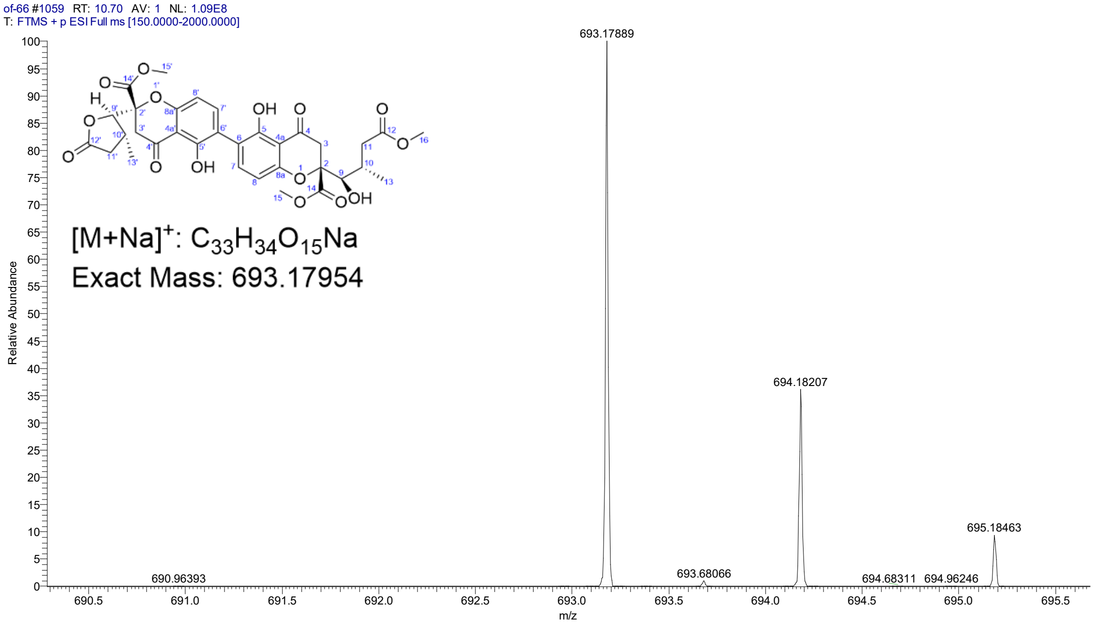

**Figure S35** $^1$H NMR spectrum of **7** (600 MHz, $CDCl_3$).

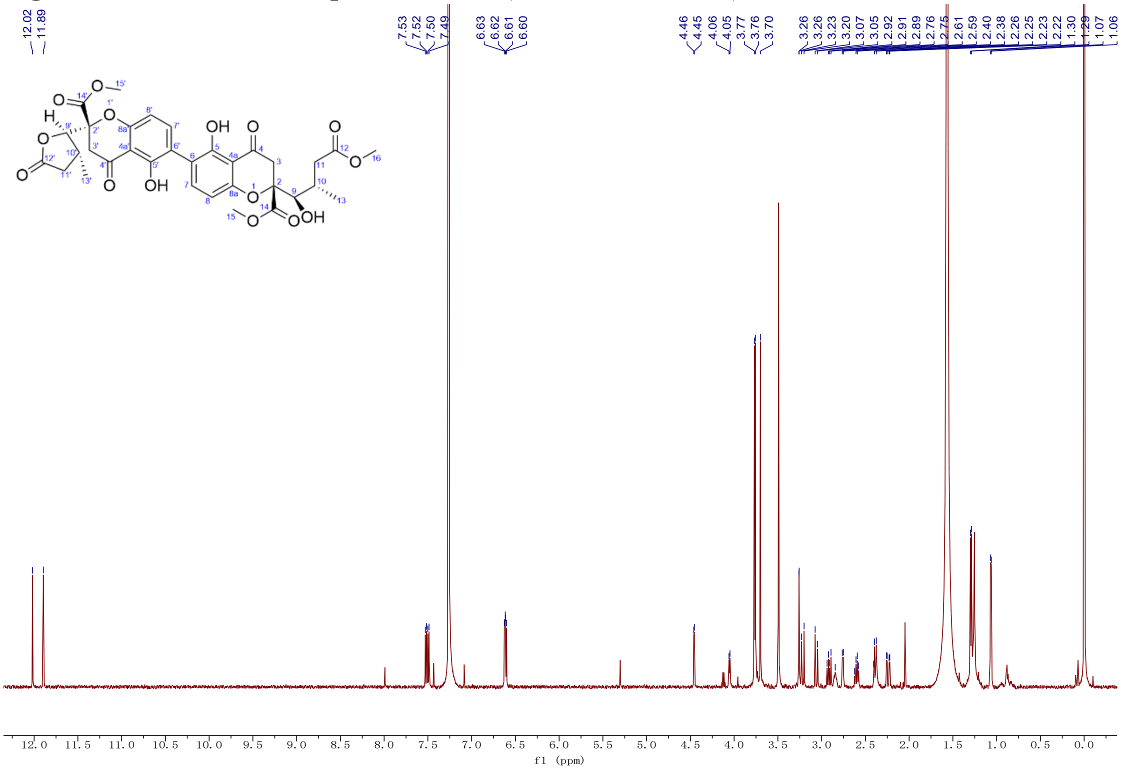

**Figure S36** $^1$H-$^1$H COSY spectrum of **7** (600 MHz, $CDCl_3$).

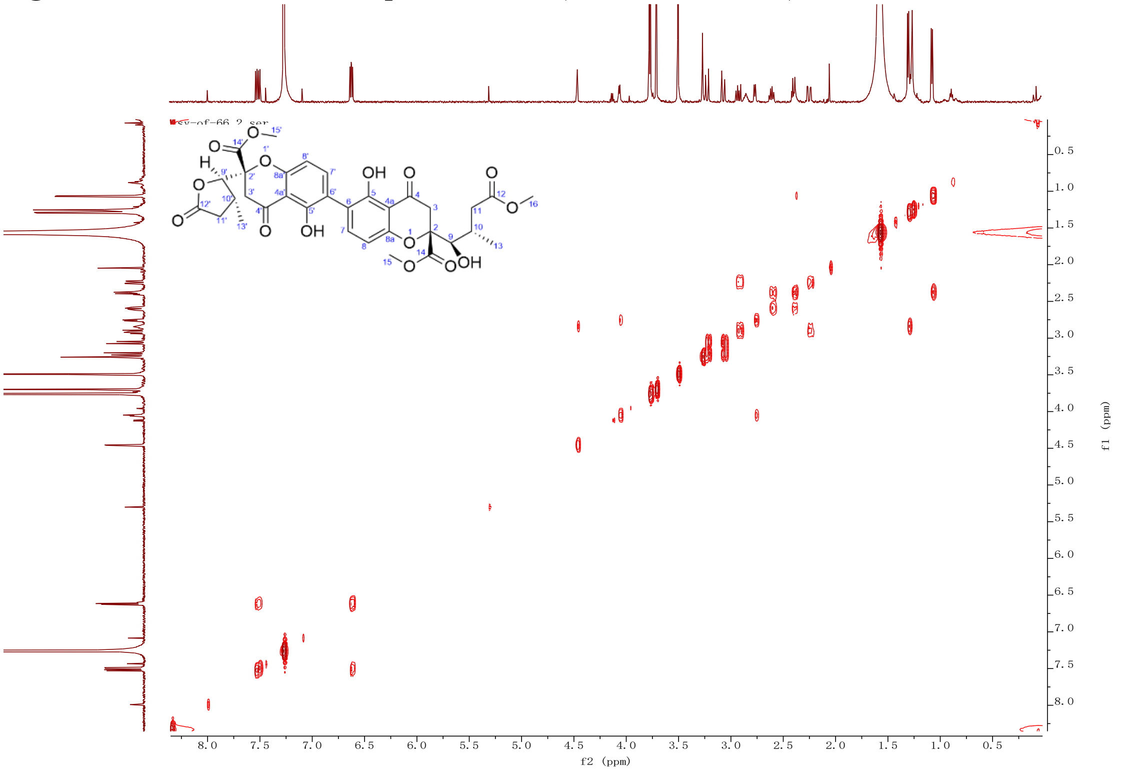

**Figure S37** HSQC spectrum of **7** (600 MHz, $CDCl_3$).

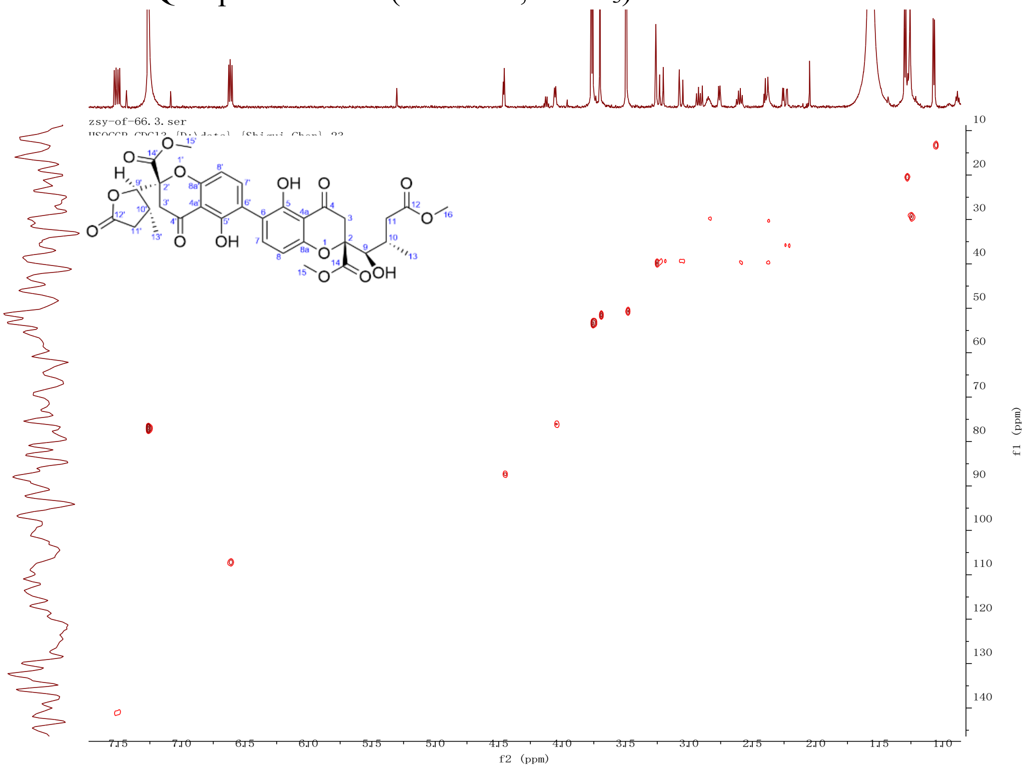

**Figure S38** HMBC spectrum of **7** (600 MHz, $CDCl_3$).

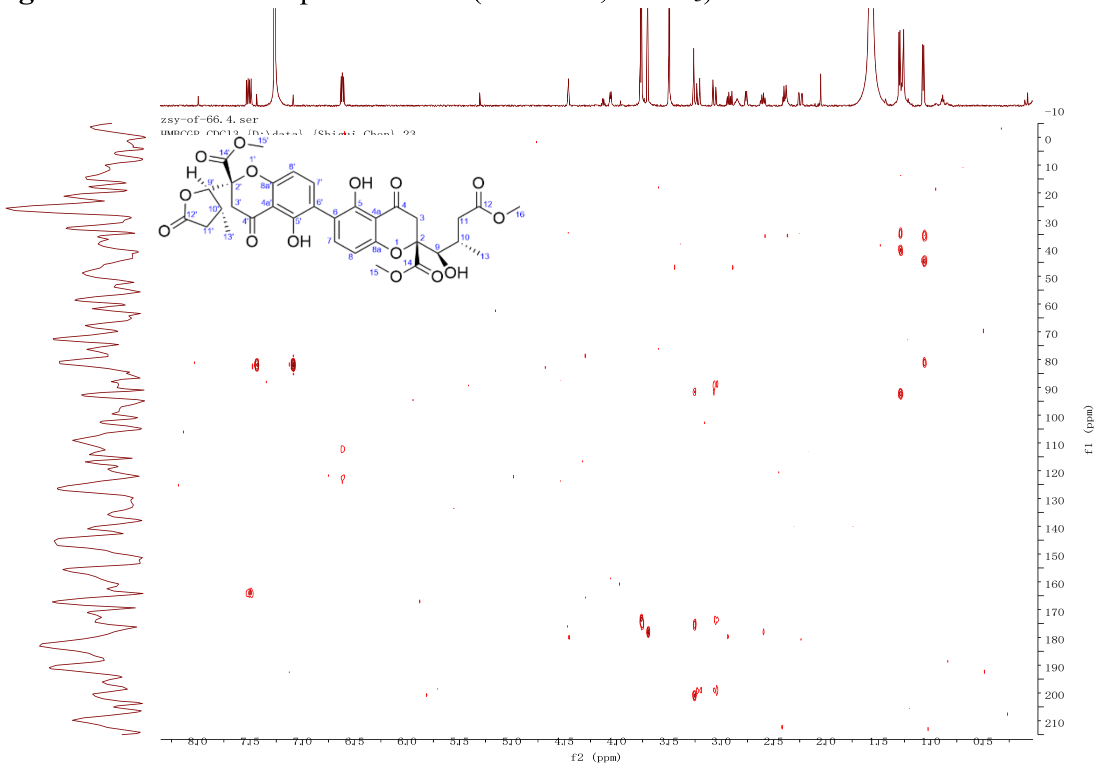

**Figure S39** NOESY spectrum of **7** (600 MHz, $CDCl_3$).

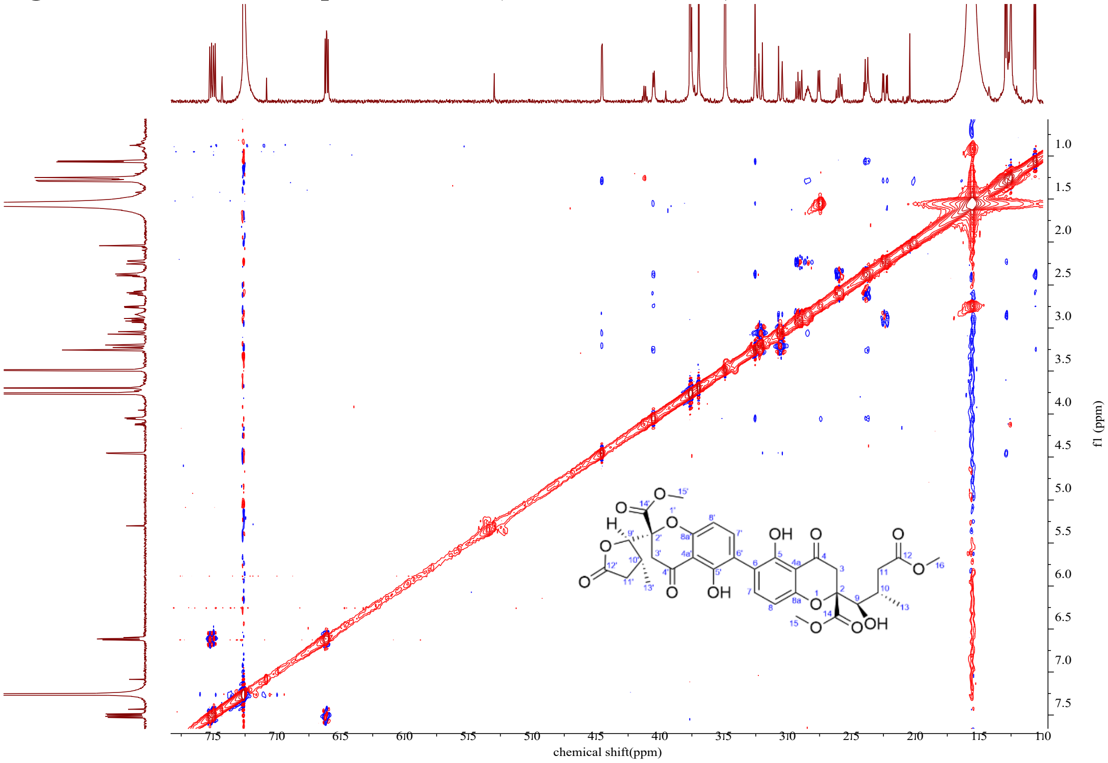


**Figure S40** Experimental ECD spectrum of **7** (MeOH).

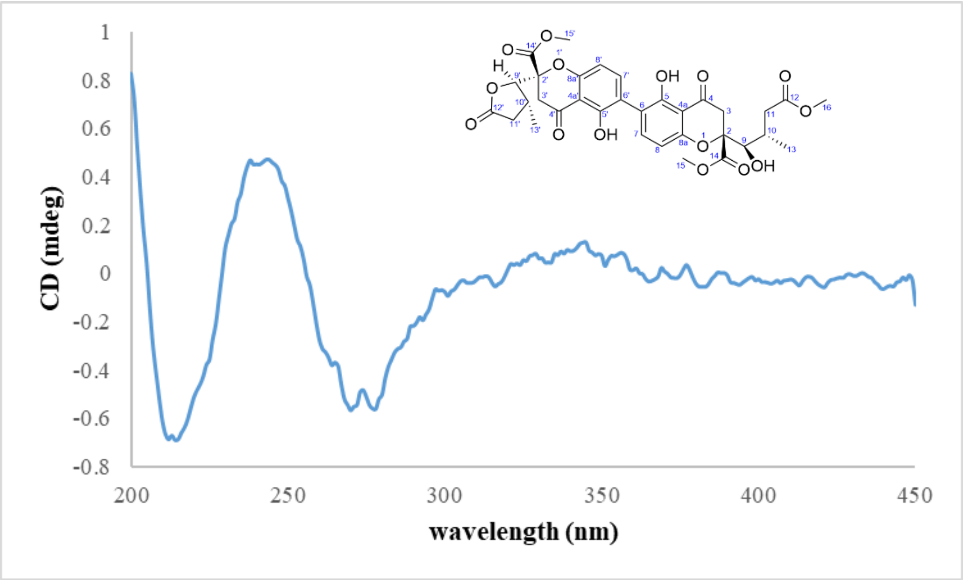

**Figure S41** HRESIMS spectrum of **8.**

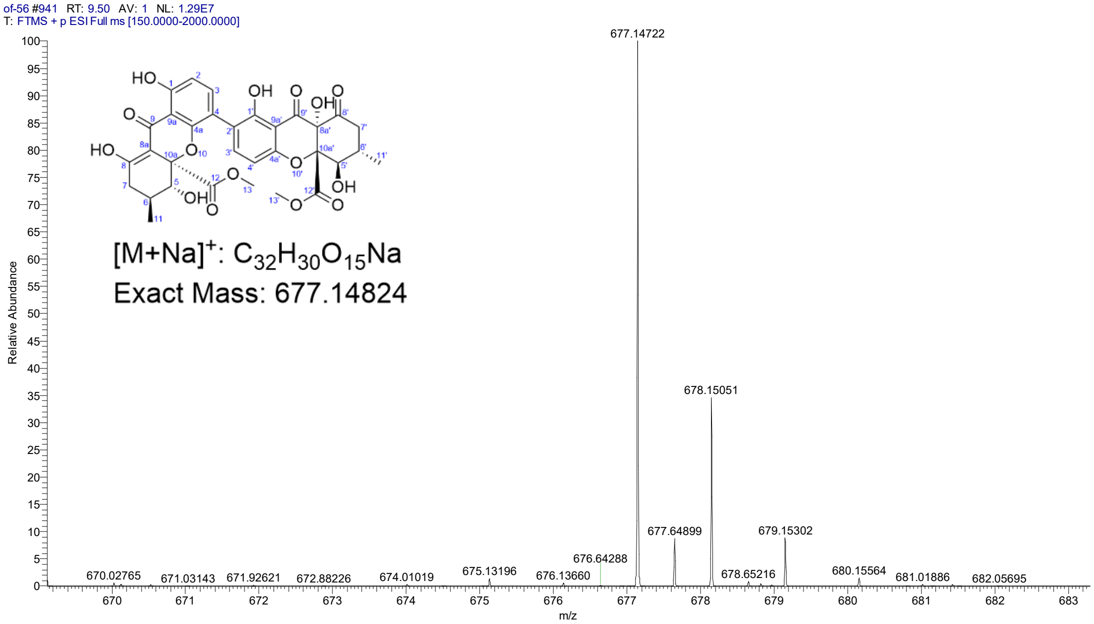


**Figure S42** $^{1}$H NMR spectrum of **8** (600 MHz, $CDCl_3$).

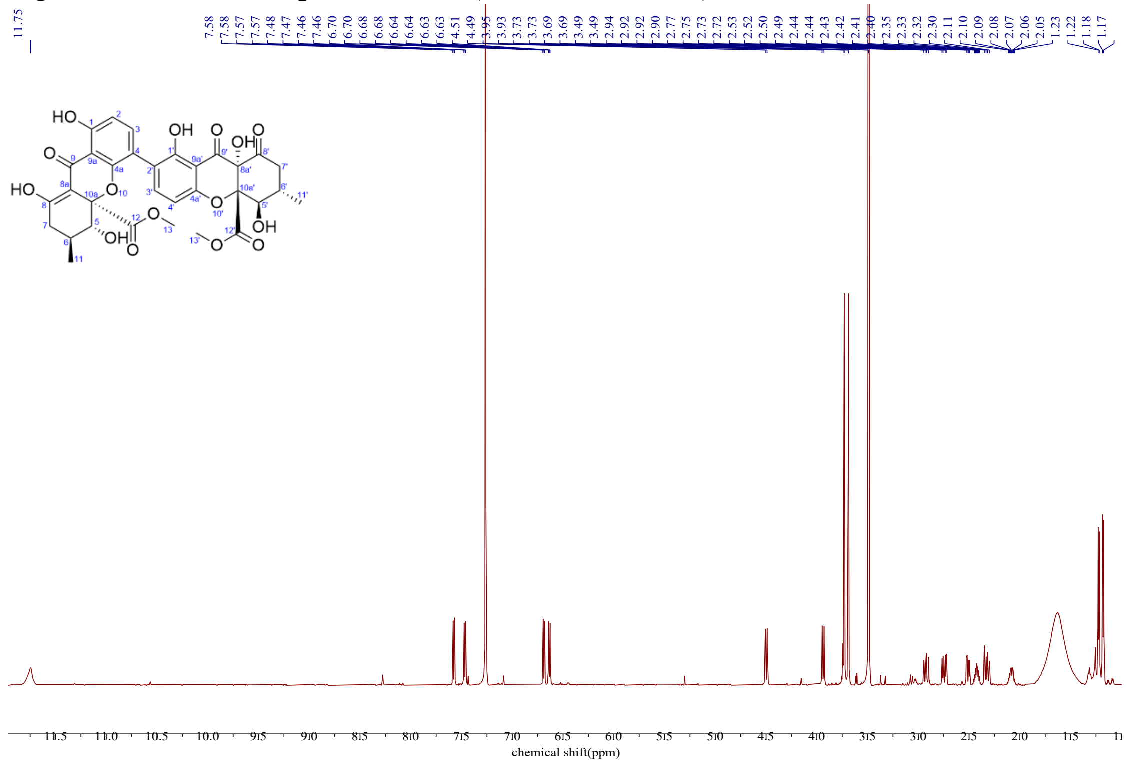

**Figure S43** $^{13}C$ NMR spectrum of **8** (150 MHz, $CDCl_3$).

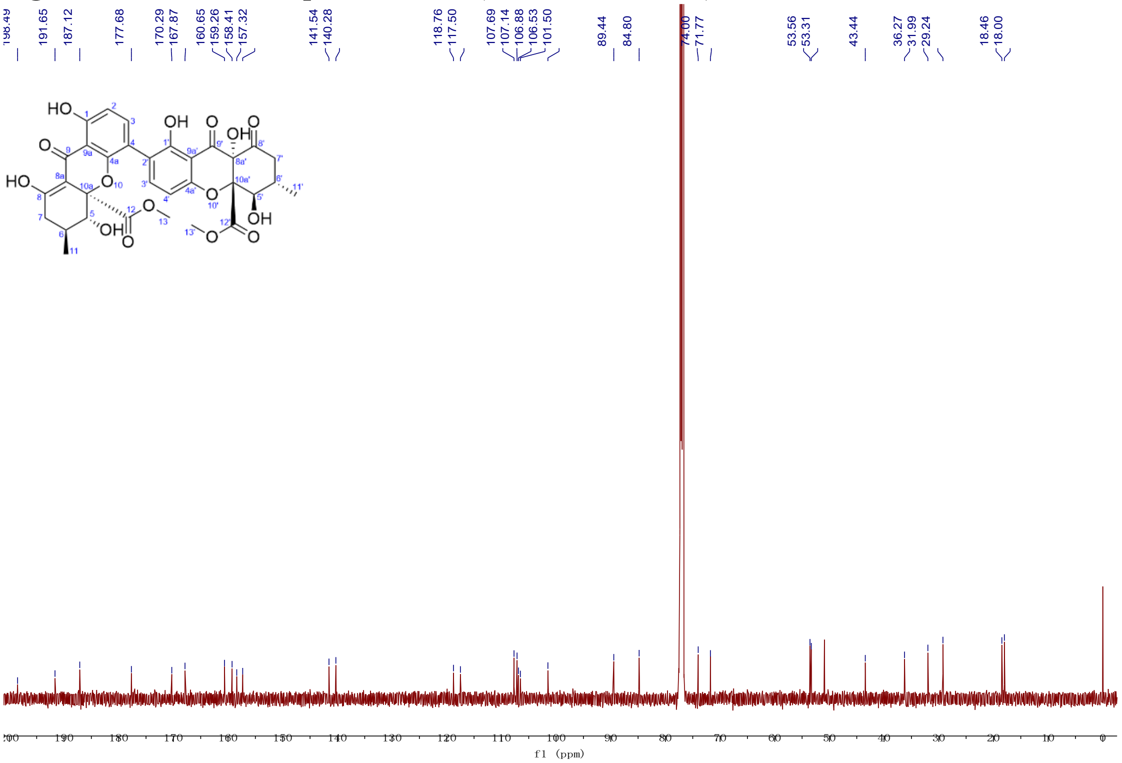

**Figure S44** $^{1}H$-$^{1}H$ COSY spectrum of **8** (600 MHz, $CDCl_3$).

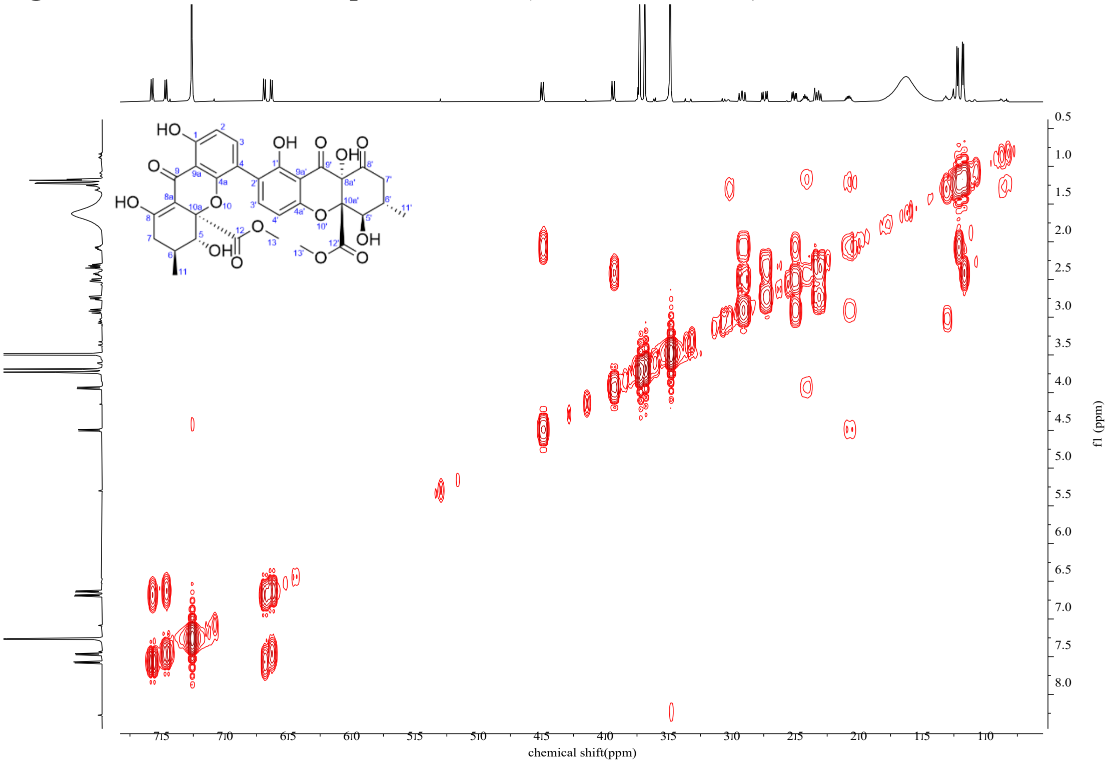

**Figure S45** HSQC spectrum of **8** (600 MHz, $CDCl_3$).

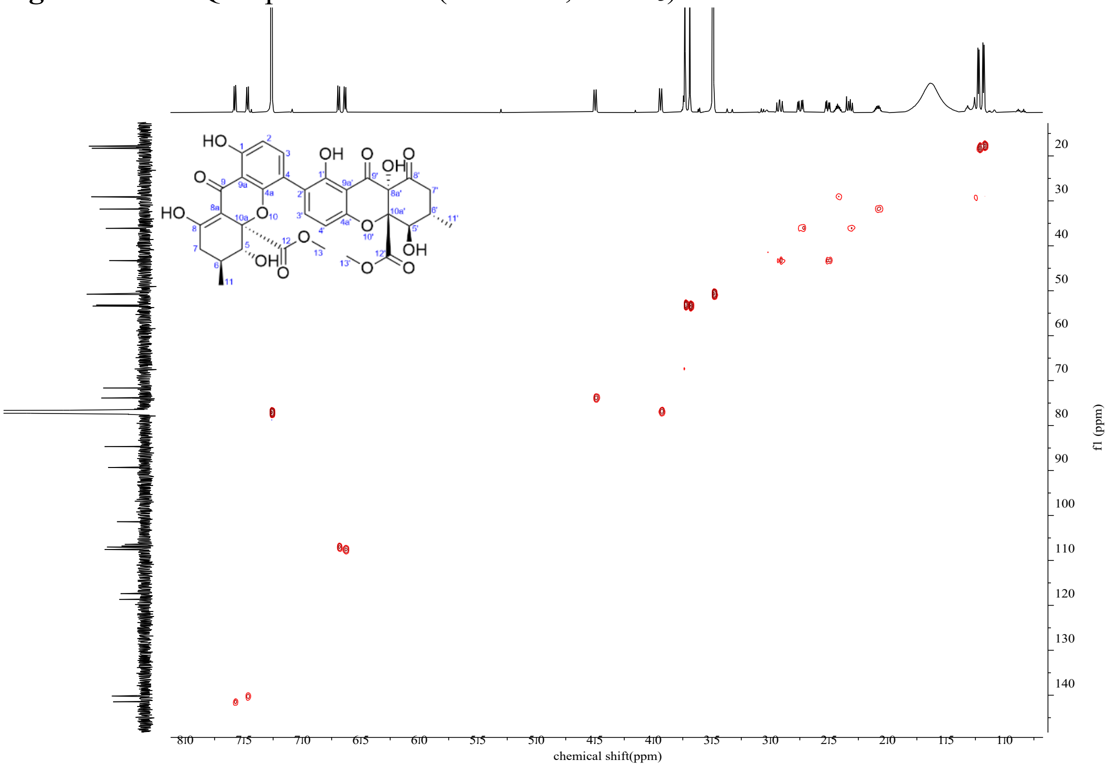

**Figure S46** HMBC spectrum of **8** (600 MHz, $CDCl_3$).

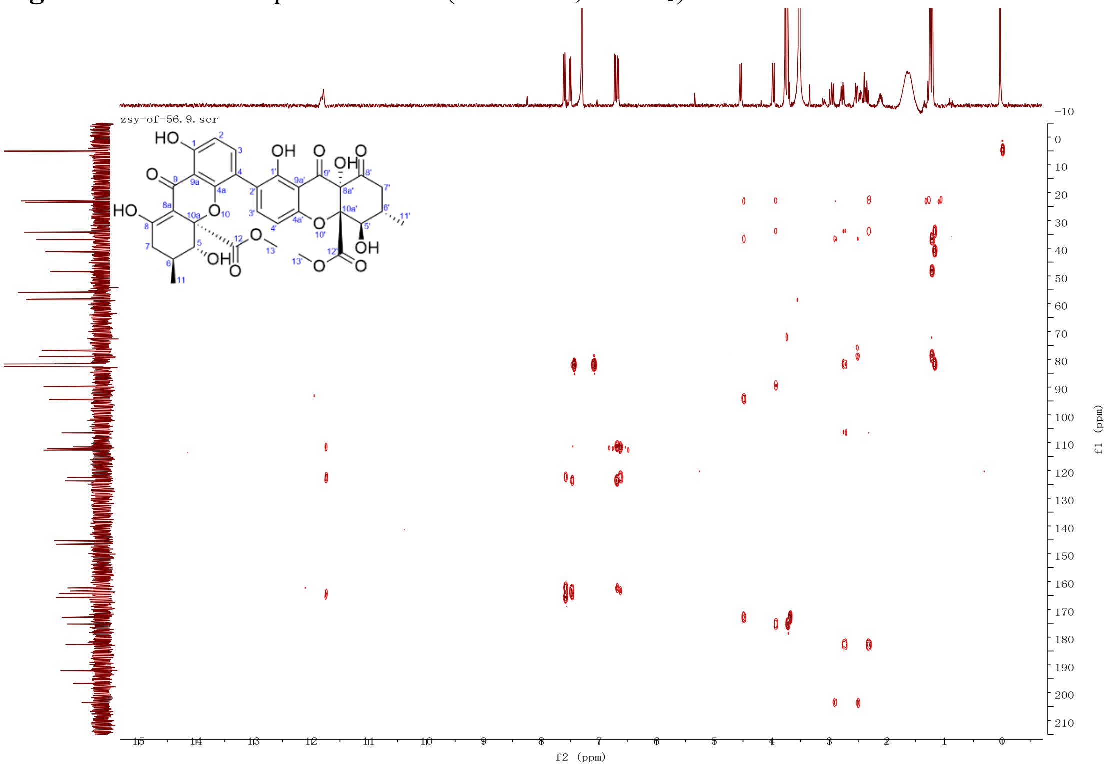

**Figure S47** NOESY spectrum of **8** (600 MHz, $CDCl_3$).

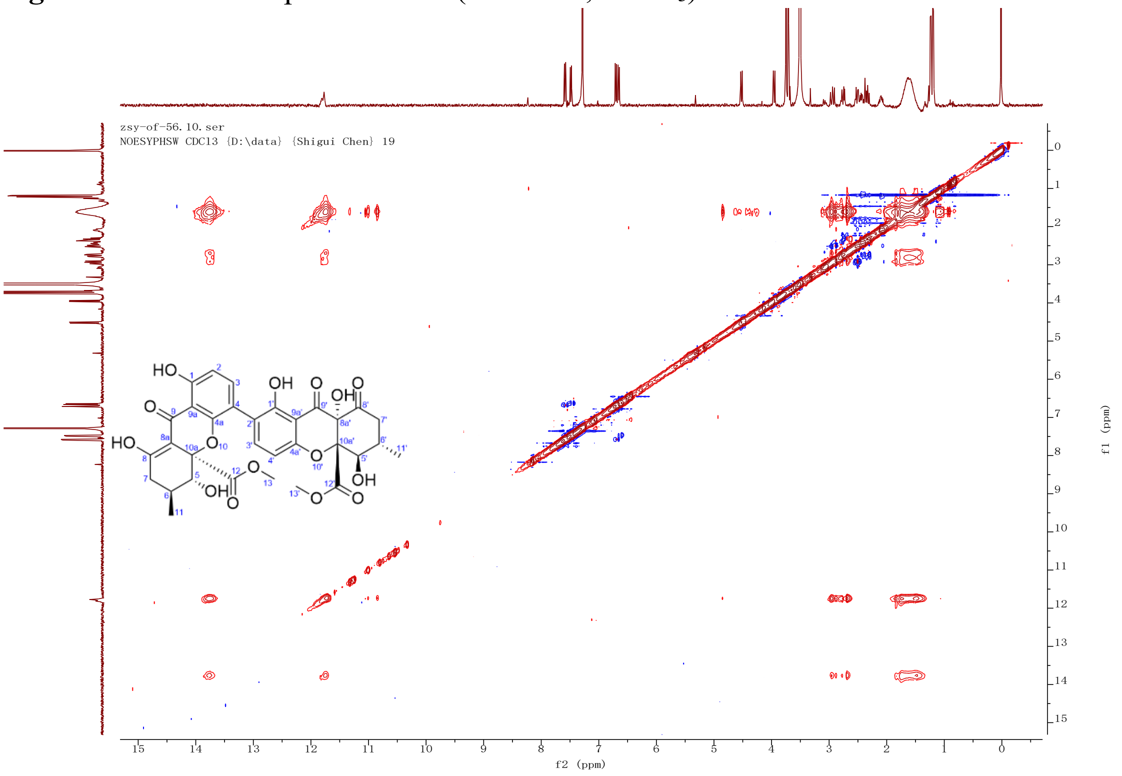


**Figure S48** Experimental ECD spectrum of **8** (MeOH).

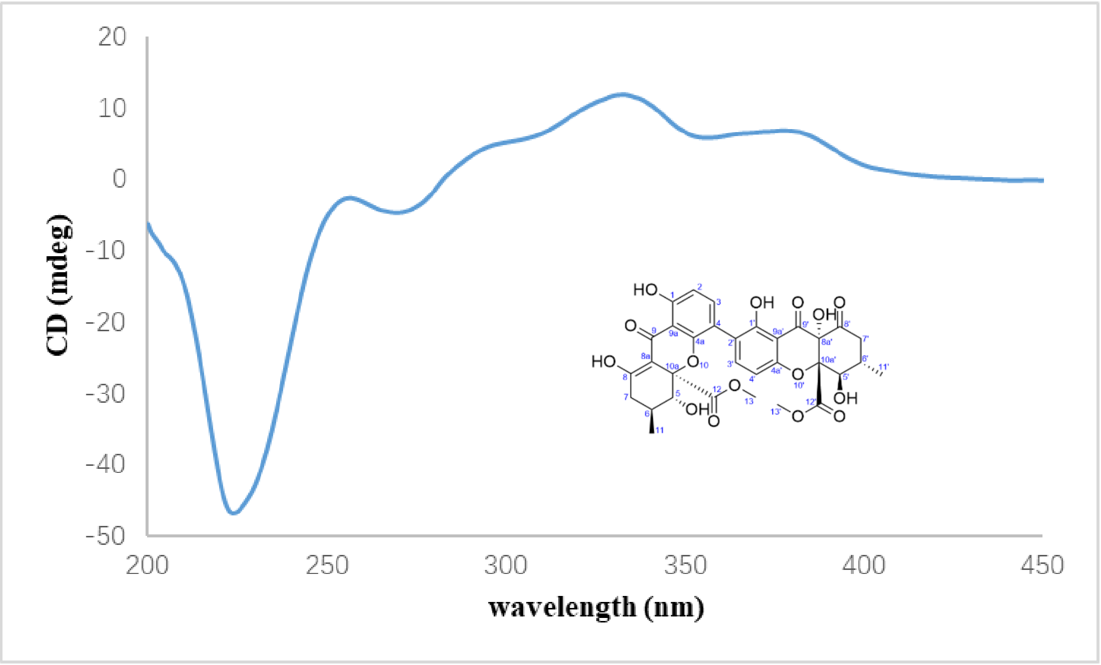

**Figure S49** HRESIMS spectrum of **9**.

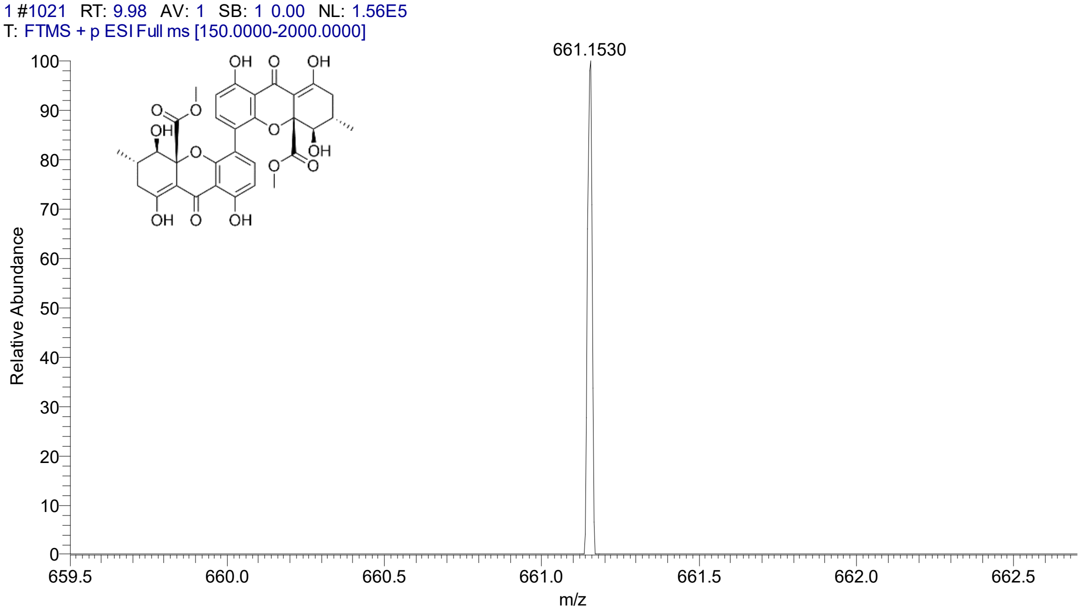


**Figure S50** $^{1}$H NMR spectrum of **9** (600 MHz, $CDCl_3$).

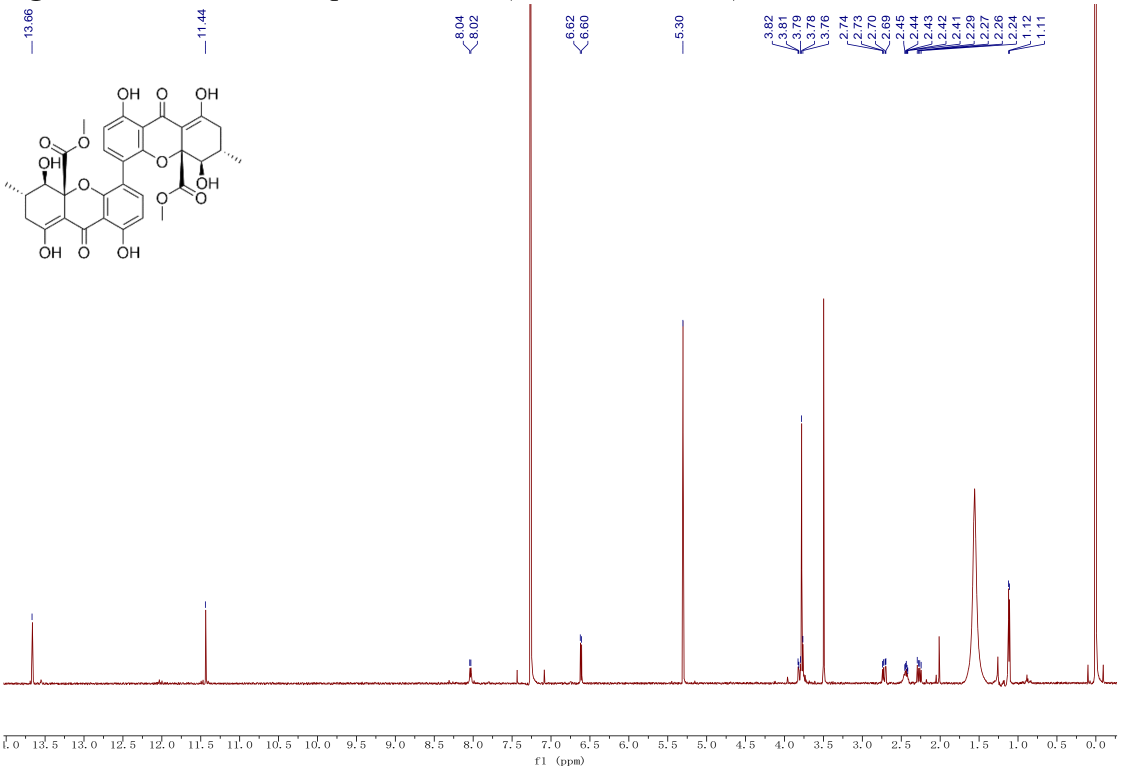

**Figure S51** Experimental ECD spectrum of **9** (MeOH).

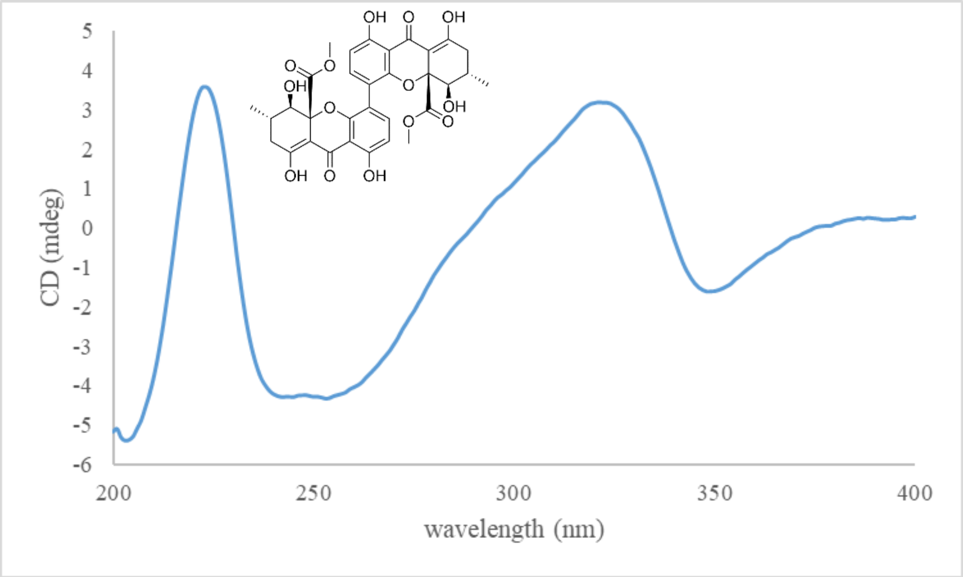


**Figure 52** Comparison of the experimental ECD spectrum with the TDDFT-predicted curves of compound **9**.

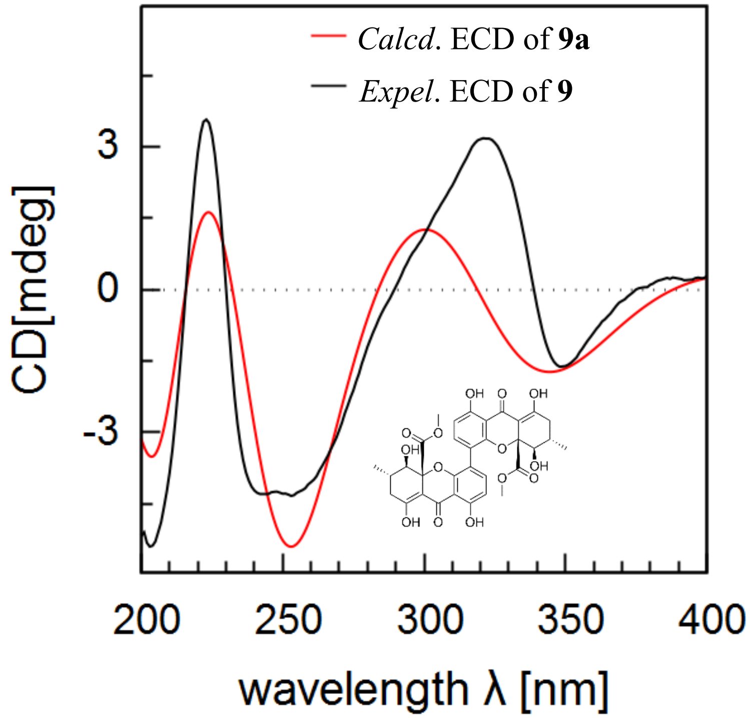

Supplement: Supplementary file 1 [file Data_Sheet_1.PDF]
